# Spatial cellular architecture predicts prognosis in glioblastoma

**DOI:** 10.1038/s41467-023-39933-0

**Published:** 2023-07-11

**Authors:** Yuanning Zheng, Francisco Carrillo-Perez, Marija Pizurica, Dieter Henrik Heiland, Olivier Gevaert

**Affiliations:** 1 Department of Medicine, Stanford Center for Biomedical Informatics Research (BMIR), Stanford University, Stanford, CA 94305 USA; 2 Department of Architecture and Computer Technology (ATC), University of Granada, Granada, 18014 Spain; 3 Internet technology and Data science Lab (IDLab), Ghent University, Technologiepark-Zwijnaarde 126, Ghent, 9052 Gent, Belgium; 4 Microenvironment and Immunology Research Laboratory, Medical Center, University of Freiburg, Freiburg, 79106 Germany; 5 Department of Neurosurgery, Medical Center, University of Freiburg, Freiburg, 79106 Germany; 6 Department of Biomedical Data Science, Stanford University, Stanford, CA 94305 USA

**Keywords:** Tumour heterogeneity, Machine learning, CNS cancer

## Abstract

Intra-tumoral heterogeneity and cell-state plasticity are key drivers for the therapeutic resistance of glioblastoma. Here, we investigate the association between spatial cellular organization and glioblastoma prognosis. Leveraging single-cell RNA-seq and spatial transcriptomics data, we develop a deep learning model to predict transcriptional subtypes of glioblastoma cells from histology images. Employing this model, we phenotypically analyze 40 million tissue spots from 410 patients and identify consistent associations between tumor architecture and prognosis across two independent cohorts. Patients with poor prognosis exhibit higher proportions of tumor cells expressing a hypoxia-induced transcriptional program. Furthermore, a clustering pattern of astrocyte-like tumor cells is associated with worse prognosis, while dispersion and connection of the astrocytes with other transcriptional subtypes correlate with decreased risk. To validate these results, we develop a separate deep learning model that utilizes histology images to predict prognosis. Applying this model to spatial transcriptomics data reveal survival-associated regional gene expression programs. Overall, our study presents a scalable approach to unravel the transcriptional heterogeneity of glioblastoma and establishes a critical connection between spatial cellular architecture and clinical outcomes.

## Abstract (web summary)

Intra-tumoral heterogeneity and cell-state plasticity contribute to the development of therapeutic resistance in glioblastoma (GBM). Here the authors use two deep learning models to predict spatial transcriptional programs and prognosis from histology images in GBM.

## Introduction

Glioblastoma (GBM) represents the most common and aggressive form of malignant tumor in the central nervous system, characterized by a low five-year survival rate of 6.8%^1^. Despite advancements in diagnostic techniques and treatment modalities, therapeutic resistance and tumor recurrence continue to challenge clinical outcomes^2^. One of the major obstacles precluding the development of effective therapeutics is tumor heterogeneity^3,4^. Malignant cells demonstrate differences in genetic lesions, epigenetic states, and gene expression profiles^5,6^. In addition, tumors from different patients have distinct cell-type compositions and spatial cellular organization. In the past decade, studies based on single-cell RNA sequencing (scRNA-seq) have guided our understanding of intra-tumoral heterogeneity^5,7–11^. GBM cells span between four major cellular states: (1) neural-progenitor-like (NPC-like), (2) oligodendrocyte-progenitor-like (OPC-like), (3) astrocyte-like (AC-like), and (4) mesenchymal-like (MES-like)^5^. Integrated analysis of GBM and normal developmental brains revealed conserved trilineage differentiation hierarchy of GBM cells that mirror normal neurodevelopment^10^.

While scRNA-seq can profile transcriptomes of thousands of cells in a single experiment, it only provides indirect inference of cell-to-cell interactions due to the loss of spatial information. In brain malignancies, cellular interactions and tumor architecture are key factors driving the clonal evolution, tumor progression and therapeutic resistance^12–15^. Recent advancements of spatial transcriptomics technologies have enabled in situ transcriptome profiling without the need for tissue dissociation. This provides a unique opportunity to decipher how malignant cells are spatially organized and interact with their immediate microenvironment. Recent studies based on spatial transcriptomics have revealed spatial localization of GBM cells with distinct transcriptional phenotypes^16–18^.

Although scRNA-seq and spatial transcriptomics enable us to decipher tumor compositions, these technologies are expensive, require specialized expertise, and are not included as a routine assay for cancer diagnosis, which restricts their clinical applications. Therefore, how cellular composition and spatial architecture contribute to patient prognosis has not been completely resolved. Compared to transcriptome profiling on the other hand, histology images are widely available and easier to obtain. In addition, the recent advancement in digital profiling of whole-slide images (WSIs) has enabled the generation of high-resolution cellular maps of tumors from large patient cohorts^19^. These technical advances have motivated studies that use deep learning to automate clinical diagnosis^20,21^, detect metastasis^22^, quantify immune-infiltrating cells^23,24^, classify cancer subtypes and predict tumor grade^25,26^. Some others used it for predictions of molecular traits, such as gene expression^27^, mutations^28^, copy number alterations^29^ and hormone receptor status^30^. Since molecular profiles are known to shape cell morphological features, we hypothesize that the transcriptional subtypes of malignant cells can be inferred from histology images, and this will enable us to computationally reconstruct cellular maps with informed transcriptional subtypes and link spatial cellular architectures to clinical outcomes.

In this study, we utilize a reciprocal approach to investigate the effect of transcriptional subtype compositions and spatial cellular organization on GBM prognosis. Firstly, we develop a deep learning model capable of predicting the transcriptional subtypes of malignant cells based on histology images. The model is trained using spatial transcriptomics data and validated in external testing cohorts. Leveraging histology images from 410 patients, we phenotypically analyze 40 million tissue spots and identify consistent associations between tumor architecture and prognosis across two independent GBM cohorts. Additionally, we train a separate deep learning model that leverages histology images to predict prognosis. Applying this model to spatial transcriptomics data lead to the identification of survival-associated regional gene expression programs. Finally, we develop a user-friendly software, named *GBM360*, that allows users to characterize tissue compositions and spatial cellular organization of new GBM cases. Although the current study focuses on GBM, the multi-modal data integration framework presented here is scalable to other diseases.

## Results

### Identifications of spatially resolved transcriptional subtypes

**Fig. 1:**
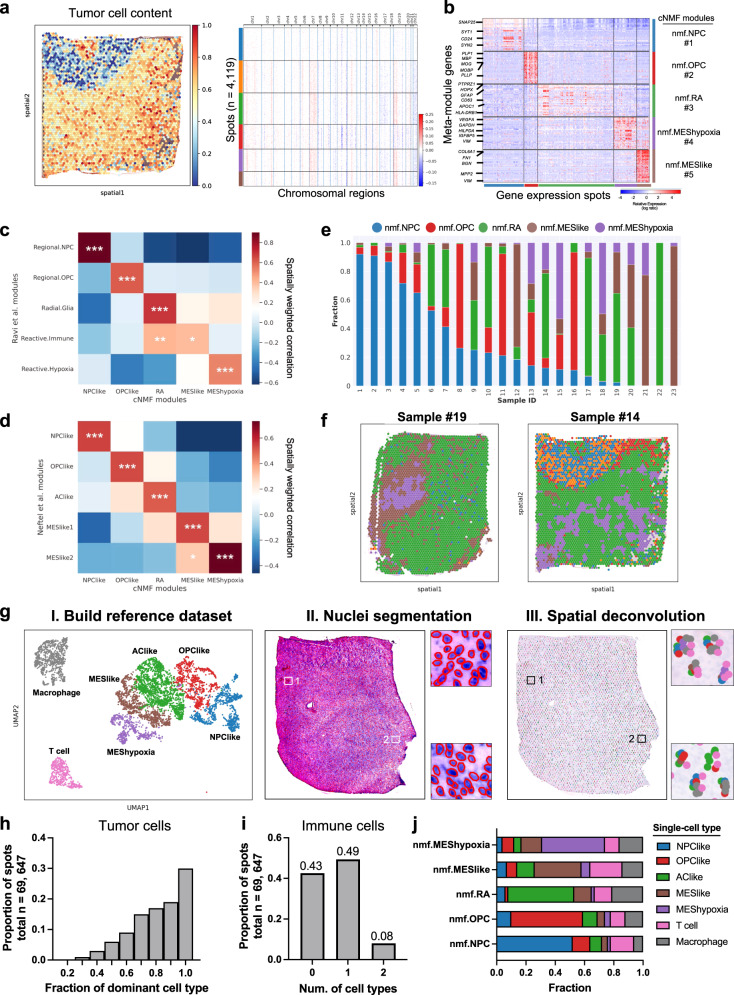
Identifications of spatial gene expression programs in GBM. **a** Heatmaps showing the tumor cell content across different spots and corresponding CNAs across different chromosomes in a representative sample. **b** Heatmap showing gene expression levels of the top 60 signatures from each cNMF module. Malignant spots (*n* = 69, 647) from all samples (*n* = 23) were grouped by the expression score of each module. **c**, **d** Heatmaps showing the average correlation coefficients (*n* = 23 samples) from spatially weighted correlation analysis between the cNMF modules (x-axis) and published modules from (**c**) Ravi et al. ^16^ and (**d**) Neftel et al. ^5^ Two-sided Wald tests were used to determine statistical significance, and *P* values were adjusted for multiple testing using the Benjamini-Hochberg procedure. *P < 0.05, **P < 0.01, ***P < 0.001. **e** Stacked bar plot showing the fractions of different transcriptional subtypes in each sample. Transcriptional subtype was determined using the top-scoring cNMF module in each spot. **f** spatial visualization showing the distribution of transcriptional subtypes in two example tumors. Spots were colored by transcriptional subtypes as indicated in panel e. **g** Pipeline for computational deconvolution of spots using single-cell RNA-seq data as reference: I. UMAP visualization of the reference single-cell RNA-seq data. Each dot represents a cell colored by the subtype; II. cell count estimation of each spot based on nuclei segmentation; III. Align cell types from the reference dataset to spots. **h** Histogram showing the fraction of dominant tumor cell type over all tumor cells in each spot (total *n* = 69, 647 spots*; n* = 23 samples). **i** The number of immune cell types in each spot (total *n* = 69, 647 spots; *n* = 23 samples). **j** The average fraction (x-axis) of each individual cell type from the single-cell RNA-seq data in spots classified by cNMF modules (y-axis). Source data are provided as a Source Data file.

To resolve the transcriptional heterogeneity of GBM within the spatial context, we performed an integrative analysis of three spatial transcriptomics datasets (Supplementary Data 1)^16,18^. The integrated dataset comprised 23 GBM samples obtained from 22 patients. Each sample contains 2500 ~4702 gene expression spots, resulting in 75,625 transcriptomes. Data preprocessing and batch-effect normalization were described in the “Methods” section. To determine the number of cells in each spot, we performed nuclei segmentation on histology images. The cell count ranged from 3 to 38, with an average count of 13 cells (Supplementary Fig. 1a). To determine genomic abnormalities of the GBM samples, we inferred copy number alterations (CNAs) using the transcriptomics profile of each spot, where data from a separate cohort of normal brain tissues (*n* = 6 tissues from 3 patients) were used as a reference^31^. Tumor samples demonstrated broad CNAs across chromosomes, including gains of Chr 6, Chr 7 and loss of Chr 8, Chr 10 and Chr14 (Fig. 1a and Supplementary Fig. 1b). Since GBM cells are highly infiltrative, each spot may contain a mixture of tumor cells and normal brain tissues. To estimate the tumor cell content within each spot, we first designated a prominent CNA event that shared across all the spots in each tumor as tumor signature CNA. The tumor cell content was then estimated based on the score of the CNA signature (“Methods”). At least three signature events were calculated in each tumor to ensure robust and unbiased estimations. We found that our approach was able to distinguish tumor regions versus histologically normal peripheral tissues (Supplementary Figs. 1c, d). Therefore, we used CNA-based estimation of tumor cell contents to filter malignant spots, while spots with low (<20%) tumor cell content were removed in our subsequent analysis.

To identify transcriptional subtypes of the malignant spots, we employed two complementary approaches. First, we performed consensus non-negative matrix factorization (cNMF)^32^ using transcriptomes from malignant spots to identify recurrent gene expression modules across the patients. Second, we performed computational deconvolution of the spots using data from single-cell RNA-seq as references. Through the cNMF analysis, we discovered five distinct meta-gene modules (Fig. 1b and Supplementary Data 2). To differentiate these modules from the published single-cell RNA-seq modules^5^, we named each of them as “nmf.x” (e.g., nmf.NPC). The first two modules were associated with neuronal lineage development and synaptic functions (Fig. 1b and Supplementary Figs. 2a, b). Module #1 was enriched with markers for neural progenitor cells (*SNAP25*, *CD24* and *SYN1*)^33^, while module #2 was strongly associated with oligodendrocyte progenitors (*PLP1*, *CNP, MBP*)^6,8^. Therefore, we designated module #1 as nmf.NPC and module #2 as nmf.OPC. Module #3 exhibited co-expression of astrocytic markers (*GFAP* and *APOC1*)^6^ and genes involved in antigen processing and inflammatory response (e.g., *HLA-DRA, B2M*, and *CD74*) (Fig. 1b and Supplementary Fig. 2c). This co-expression pattern likely reflects the reactive transformation of astrocytes^34–36^. Therefore, we named module #3 as nmf.RA (reactive astrocytes). The remaining two modules, #4 and #5, were enriched with mesenchymal (MES)-related genes, such as *VIM* and *COL6A1* (Fig. 1b). Module #4 demonstrated enrichment in glycolytic process (*GAPDH*, *PGK1*, *LDHA*) and hypoxia response (*VEGFA*, *HILPDA*, *ADM*) (Supplementary Fig. 2d), hence named as nmf.MES-hypoxia. On the other hand, module #5 was enriched with genes encoding extracellular matrix (*COL6A1*, *FN1*, *MMP9*), but lacked hypoxia signatures, and was designated as nmf.MES-like (Supplementary Fig. 2e).

To assess how the cNMF modules were related to published transcriptional modules in GBM, we performed spatially weighted correlation analyses. We first compared each cNMF module to the modules defined in a spatial transcriptomics study by Ravi et al. ^16^ As expected, we observed strong correlations between the nmf.NPC and nmf.OPC modules with the Regional.NPC and Regional.OPC modules, respectively (Fig. 1c, *P* < 0.001). The nmf.RA correlated with both the Radial.Glia (*P* < 0.001) and Reactive.Immune (*P* < 0.01) modules. This overlap is expected due to the close relationship identified between the Radial.Glia and Reactive.Immune modules in the original study^16^. The nmf.MES-like and nmf.MES-hypoxia modules were significantly correlated with the Reactive.Immune and Reactive.Hypoxia module, respectively (Fig. 1c). To provide additional validation, we further compared our modules to those defined from a single-cell RNA-seq study by Neftel et al. ^5^ The nmf.NPC, nmf.OPC, and nmf.RA modules demonstrated strong correlations with the NPClike, OPClike and AClike modules, respectively (Fig. 1d). Similarly, the nmf.MES-like and nmf.MES-hypoixa modules were strongly correlated with the MESlike1 and MESlike2 modules, with the nmf.MES-hypoixa module specifically correlated with the MESlike2 module. Notably, the MESlike2 module identified by Neftel et al. was also enriched with hypoxia-response genes, demonstrating the strong relationship between the cNMF modules and those derived from single-cell RNA-seq. By analyzing the transcriptional subtypes of spots determined by the top-scoring cNMF module, we discovered that each tumor harbored multiple transcriptional subtypes (Fig. 1e). Spots of different subtypes were localized within distinct spatially segmented regions (Fig. 1f). These observations highlight the transcriptional diversity and spatial heterogeneity within GBM tumors.

Next, we performed deconvolution analysis to estimate the fraction of different GBM cell types with the malignant spots using data from single-cell RNA-seq (Fig. 1g and **“Methods”**). To capture both tumor cells and immune cells, we integrated three single-cell RNA-seq datasets as references: GSE131928, GSE163108, and GSE84465 (Supplementary Data 1)^5,11,37^. For tumor cells, we included four transcriptional subtypes from the reference: (1) NPClike, (2) OPClike, (3) AClike, (4) MESlike. The MESlike cells were further classified into hypoxia-dependent (MEShypoxia) and hypoxia-independent (MESlike) groups based on the expression of hypoxia-response genes (e.g., *HILPDA*, *VEGFA*) and glycolytic genes (e.g., *GAPDH, LDHA*)^5^. For immune cells, we focused on T cells and macrophages. Details of the deconvolution analysis and validation of the results can be found in the “Methods” section (Supplementary Figs. 1e, f). Based on our deconvolution analysis, we found that the composition of tumor cells was relatively homogenous within individual spots. In most spots, the dominate tumor cell type accounted for over 70% of all tumor cells (Fig. 1h). Notably, approximately 30% of the spots consisted exclusively of one tumor cell type (Fig. 1h). Analysis of the immune cell distributions showed that 49% of the spots contained one immune cell type, and approximately 8% of the spots consisted of a mixture of both T cells and macrophages (Fig. 1i). Overall, the spots classified as nmf.NPC, nmf.OPC, nmf.RA, nmf.MES-like, nmf.MES-hypoxia were enriched with NPClike, OPClike, AClike, MESlike and MEShypoxia cells, respectively (Fig. 1j). In addition, the nmf.RA spots had increased macrophage infiltration compared to the other spots, while the nmf.MESlike spots had increased proportions of T cells. These results demonstrated that a single spot was typically dominated by one tumor cell type, while the tumor cells were frequently mixed with immune cells.

### Transcriptional subtypes can be predicted from histology images

**Fig. 2:**
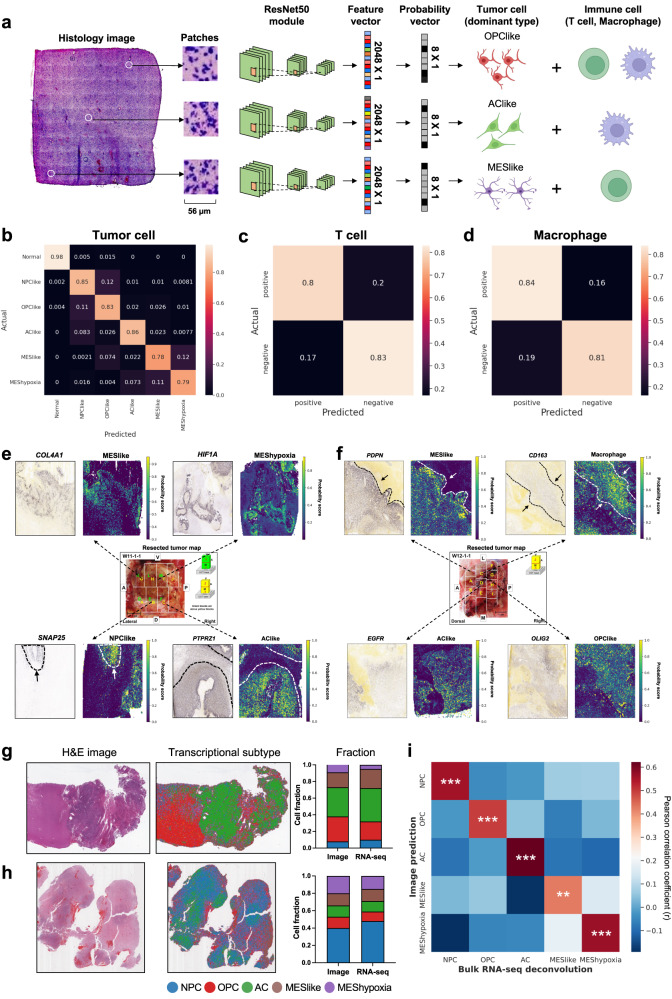
Development and validation of GBM-CNN for spatially resolved transcriptional subtype prediction. **a** Architecture of GBM-CNN. Histology images were cropped to extract patches corresponding to each spot. Each patch was then transformed into a feature vector (2048 $\times$ 1) using a ResNet-50 module. Subsequently, each feature vector was mapped to a probability vector (8 $\times$ 1) through a fully connected layer. The cell-type cartoons were created with BioRender.com. **b** Confusion matrix showing the classification performance of GBM-CNN in predicting the dominant tumor cell type. Predictions from all folds (n = 23) were averaged into a single matrix. **c**, **d** Confusion matrices showing the classification performance of GBM-CNN in predicting the presence of (**c**) T cell and (**d**) macrophage. **e**, **f** Alignment of ground truth gene expression signals obtained from in situ RNA hybridization and the predicted probability scores of individual cell types in matched histological sections. Examples from two different tumors were presented. **g**, **h** H&E images and the predicted distribution of transcriptional subtypes in two tumors from the TCGA cohort. Bar graphs depict transcriptional subtype proportions derived from the image prediction versus bulk RNA-seq deconvolution. **i** Heatmap of Pearson correlation coefficient showing the agreement between transcriptional subtype proportions derived from our image predictions versus those estimated from bulk RNA-seq deconvolution (*n* = 166 patients). *P* values were determined using the two-sided Pearson correlation test and were adjusted by the Benjamini-Hochberg procedure. **P < 0.01, ***P < 0.001. Source data are provided as a Source Data file.

Since gene expression features are known to shape cell morphology, we hypothesized that the cell-type distribution can be inferred from histology images. We developed GBM-CNN, a convolution neural network for image classification (Fig. 2a). The input to GBM-CNN were patches extracted from hematoxylin-eosin (H&E)-stained histology images. The edge length (56 $\mu m$) of one patch was roughly equal to the diameter (55 $\mu m$) of one gene expression spot in spatial transcriptomics. The output was cell types present in each patch. We formulated the problem as a multi-label classification task. For predicting tumor cells, since each spot was predominately occupied by only one cell type (Fig. 1h), we aimed at predicting the dominant type of tumor cells in each patch. For immune cells, since both T cells and macrophages were frequently mixed with tumor cells (Fig. 1i, j), we included them as independent labels. To assess the performance of GBM-CNN, we carried out leave-one-out cross-validation (LOOCV) using data from the spatial transcriptomics cohort (n = 23 tumors; n = 69,647 spots). In each iteration, the model was trained on spots from 22 tumors, while spots from the remaining tumor (n = 1) were reserved for held-out validation. To prevent overfitting, the model architecture and associated hyperparameters remained consistent across all iterations (“Methods”). After each iteration, we evaluated the model’s performance on the validation sample. For predicting tumor cells, the F1 score was 0.86, and the standard deviation (SD) was 0.15 (Fig. 2b). The area under the receiver operating characteristic curve (AUROC) was 0.93 and the SD was 0.05 (Supplementary Fig. 3a). Spatial visualization showed that the model accurately predicted the distribution of dominant cell types in even the most heterogenous tumors within the cohort (Supplementary Fig. 3b). To assess whether the dominant tumor cell type correlated with any histological features, we extracted the color values of each image channel, along with the texture and histogram features from the H&E images (“Methods”). Our analysis showed that the extracted image features varied across different samples (Supplementary Fig. 3c). However, when we performed the same analysis on the feature vector (2048 $\times$ 1) extracted from the last layer of the ResNet50 module (Fig. 2a), we observed a strong correlation between the resulting clusters and the transcriptional subtypes in the validation samples (Supplementary Fig. 3d). These results indicated that GBM-CNN learned latent representations of the transcriptional subtypes beyond raw histological features.

We next assessed the performance of GBM-CNN in predicting immune cells. Since T cells and macrophages were treated as independent labels, we assessed the sensitivity and specificity for each cell type separately (Fig. 2c, d). For predicting T cells, the sensitivity was 0.80 (SD: 0.08), specificity was 0.83 (SD: 0.05), and AUROC was 0.80 (SD: 0.08) (Fig. 2c and Supplementary Fig. 3e). For predicting macrophages, the sensitivity was 0.84 (SD: 0.09), specificity was 0.81 (SD: 0.07), and AUROC was 0.89 (SD: 0.11) (Fig. 2d and Supplementary Fig. 3f). These results demonstrated that the GBM-CNN was able to accurately predict the subtypes of tumor cells and the presence of immune cells from histology images.

To test whether the classification performance of GBM-CNN can be generalized to external patient cohorts, we applied the image model to whole-slide images (WSIs) from the IvyGap cohort (*n* = 184 slides from *n* = 8 patients)^38^ and the TCGA-GBM cohort (*n* = 693 slides from 312 patients)^39^. The IvyGap cohort included data from in-situ RNA hybridization for 343 GBM-related genes and their adjacent H&E-stained histology sections. We expected that tumor cells assigned with a specific transcriptional subtype from GBM-CNN should have high messenger RNA (mRNA) expression levels (i.e., ground truth) for the corresponding cell-type signatures. As shown in Fig. 2e, f, tumor regions classified as “MESlike” showed high mRNA expression levels of the MES-related signatures, such as *COL4A1*, *PDPN*. In addition, regions classified as “MES-hypoxia” aligned with the expression of *HIF1A*. Similarly, regions classified as “NPC-like” and “OPC-like” were associated with the expression of *SNAP25* and *OLIG2*, respectively. Regions classified as “AC-like” displayed elevated expression of *PTPRZ1* and *EGFR*, and regions with macrophage infiltration aligned with the expression of *CD163* (Fig. 2f).

To further validate GBM-CNN, we predicted the transcriptional subtypes of tumor cells using WSIs from the TCGA-GBM cohort^39^. Each tumor was composed of three to five transcriptional subtypes, with 76.2% of the tumors consisting of all five subtypes (Fig. 2g, h and Supplementary Fig. 3g). Although spatial transcriptomics data were not available for this cohort, we estimated transcriptional subtype proportions of each tumor by computationally deconvoluting the matched bulk RNA-seq data (“Methods”). Since bulk RNA-seq and histology images are independent modalities, we could validate our image model by comparing the subtype composition estimated from RNA-seq deconvolution versus our image predictions. Remarkably, we observed a significant correlation between the transcriptional subtype proportions estimated from bulk RNA-seq deconvolution and those predicted by our image model (Fig. 2g, h, i). These results indicated that the transcriptional subtypes of malignant cells can be predicted from histology images with GBM-CNN.

### Associations between the transcriptional subtype composition and prognosis

To assess how the predicted transcriptional subtype composition was associated with prognosis, we used diagnostic slides from patients of the TCGA cohort^39^ as the discovery cohort, and slides from the CPTAC cohort^40^ as the validation cohort (Supplementary Data 1). Since the absolute size for the resected tumor region varied across patients, the downstream analysis of tissue compositions may lead bias to tumors with large resections. To overcome this potential sampling bias, we implemented two strategies. First, we ranked the tumors in each cohort based on their number of patches (indicating tissue size) and removed the bottom 5% tumors with the smallest number of patches. Second, we included gender, age, IDH status, and tissue size as covariates in our Cox regression analysis. Following this rigorous filtering strategy, we obtained a final set of 693 slides (*n* = 312 patients) in the TCGA cohort and 227 slides (*n* = 98 patients) in the CPTAC cohort. Our multivariate Cox regression analysis showed that samples with a high proportion of patches classified as MES-hypoxia were associated with a worse prognosis (Table 1). In the TCGA cohort, the hazard ratio (HR) for the MES-hypoxia subtype was 2.06 (*P* = 0.008), and in the CPTAC cohort was 2.23 (*P* = 0.01). Moreover, the proportion of NPC-like subtype was associated with a better prognosis in both cohorts, although these associations did not reach statistical significance.

**Table 1:** Cox regression analysis showing the effect of subtype proportions on prognosis

	TCGA (*n* = 312 patients)			CPTAC (*n* = 98 patients)		
Transcriptional subtype	HR	95% Cl	*P*	HR	95% Cl	*P*
NPC-like	0.72	0.43-1.21	0.22	0.69	0.25–1.90	0.48
OPC-like	0.80	0.46-1.42	0.45	1.54	0.75–3.19	0.24
AC-like	1.25	0.95-1.36	0.80	0.78	0.65-1.20	0.87
MES-like	0.88	0.47-1.62	0.68	0.89	0.76-1.02	0.81
MES-hypoxia	2.06	1.04–4.06	0.008**	2.23	1.08–3.49	0.01*

HR: hazard ratio; CI: confidence interval. *P < 0.05; **P < 0.01.

### Associations between spatial cellular architecture and prognosis

**Fig. 3:**
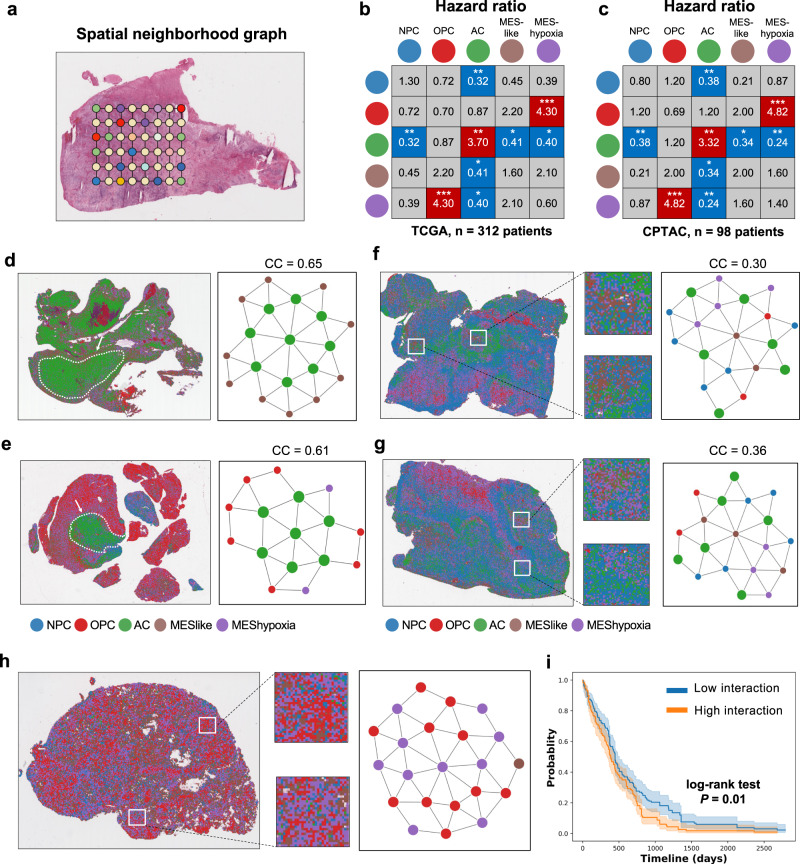
Associations between spatial cellular architecture and prognosis. **a** Schematic representation of a spatial neighborhood graph. Each patch represents a node and connections between patches are edges. **b**, **c** Hazard ratio (HR) for frequency of transcriptional subtype interactions and prognosis using data from the (**b**) TCGA (n = 312 patients) and (**c**) CPTAC (*n* = 98 patients) cohorts. Statistical significance was determined using multivariate Cox regression analysis, and significant associations were highlighted by red for HR > 0 and blue for HR < 0. *P < 0.05, **P < 0.01, ***P < 0.001. **d**, **e** Representative tumor samples with high clustering coefficient (CC) of the AC-like subtype. Spots were colored by transcriptional subtypes. Abstractive networks demonstrate tumor regions characterized by clusters of AC-like spots, as indicated by white arrows. **f**, **g** Representative tumor samples with low clustering coefficient (CC) of the AC-like subtype. Spots were colored by transcriptional subtype. Abstractive networks highlighted the interactions between the AC-like subtype and other subtypes, such as NPC-like, MES-like and MES-hypoxia. **h** Representative tumor sample with a high frequency of interaction between the OPC-like and MES-hypoxia subtype. **i** Kaplan-Meier survival curves of TCGA patients with high (*n* = 156) and low (*n* = 156) interactions between the OPC-like and MES-hypoxia subtypes. Error bands represent confidence intervals for the estimated survival probabilities, and the survival curves are compared with the log-rank test (*P* = 0.01). Source data are provided as a Source Data file.

The results presented so far have linked transcriptional subtype compositions to patient prognosis. However, since spatial organization and cellular interactions play critical roles in driving clonal evolution, tumor progression and therapeutic resistance^12–15^, we sought to assess how the spatial distribution of malignant cells contributes to prognosis. To characterize the spatial cellular organization, we first constructed a spatial neighborhood graph to represent cell communities within each tumor (Fig. 3a). In this graph, each patch was a node and edges represented direct connections between patches. The phenotype of each node was the predicted transcriptional subtype represented by the dominant tumor cell type in each patch. To ensure unbiased exploration of the spatial cellular organization and its association with prognosis, we included subtype proportions as covariates in our survival analysis.

We first assessed how the frequency of interactions between different transcriptional subtypes contributed to prognosis. Our results revealed that an increased connectivity between AC-like subtypes corresponded to an increased risk (Fig. 3b, c and Fig. 3d, e, TCGA: HR = 3.70, *P* < 0.01; CPTAC: HR = 3.32, *P* < 0.01). Conversely, when the AC-like subtype was connected with other subtypes, such as NPC-like, MES-like, and MES-hypoxia, the risk was decreased (Fig. 3b, c and Fig. 3f, g). To further confirm these results, we examined the clustering coefficient, which indicates the degree to which the same transcriptional subtype clusters together in the spatial neighborhood graph (“Methods”). We found that a higher clustering coefficient for the AC-like subtype was associated with a poorer prognosis (Table 2, TCGA: HR = 3.82, *P* = 0.004; CPTAC: HR = 3.96, *P* = 0.009). Notably, the proportion of the AC-like subtype alone was not a significant predictor of prognosis (Table 1), highlighting the value of spatial relationships over abundance alone.

**Table 2:** Cox regression analysis showing the effect of clustering coefficient on prognosis

	TCGA (*n* = 312 patients)			CPTAC (*n* = 98 patients)		
Transcriptional subtype	HR	95% Cl	*P*	HR	95% Cl	*P*
NPC-like	1.02	0.50-1.35	0.44	0.79	0.27–2.35	0.68
OPC-like	0.81	0.69-1.21	0.37	0.64	0.20–2.04	0.45
AC-like	3.82	1.28–4.86	0.004**	3.96	1.54–5.02	0.009**
MES-like	1.61	0.83–3.10	0.16	1.04	0.41–2.62	0.93
MES-hypoxia	0.51	0.24-1.20	0.12	0.97	0.52-1.78	0.29

*HR* hazard ratio, *CI* confidence interval. **P < 0.01.

Furthermore, our analysis revealed that a higher interaction between the OPC-like and MES-hypoxia subtypes was strongly associated with a poorer prognosis in both cohorts (Fig. 3b, c and Fig. 3h, i, TCGA: HR = 4.30, *P* < 0.001; CPTAC: HR = 4.82, *P* < 0.001). Overall, these findings underscored the significance of spatial interactions between transcriptional subtypes in affecting patient prognosis.

### In situ identifications of gene expression markers associated with prognosis

**Fig. 4:**
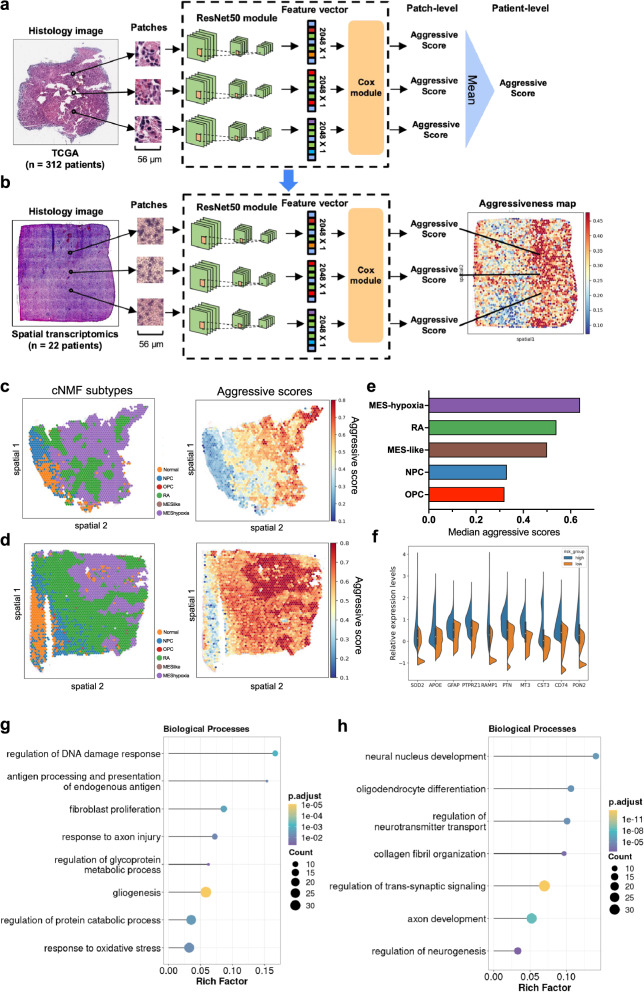
In situ identifications of gene expression markers associated with prognosis. **a** A deep-learning model was trained on whole slide images from the TCGA cohort to predict patient prognosis. H&E-stained histology images were cropped into 56μm $\times$ 56μm patches. Each patch was converted to a feature vector (2048 $\times$ 1) using a ResNet-50 module. The feature vectors were then mapped to an aggressive score through a Cox regression module. The aggressive scores of each patient were averaged for validation. **b** Using the trained image model from panel (**a)** to predict aggressive scores for spots in spatial transcriptomics. **c**, **d** Visualization of transcriptional subtypes and the predicted aggressive scores in two tumors from the spatial transcriptomics cohort. Aggressive scores were normalized within each sample using min-max normalization. **e** Bar plot of median aggressive scores for malignant spots. Aggressive scores from all tumors (*n* = 23) were pooled together and normalized using min-max normalization. **f** Violin plot of mRNA expression levels for genes upregulated in tumor regions assigned with high aggressive scores (blue) versus those with low scores (yellow). The top 10,000 spots from each group were shown. Boxes within the violins represent the interquartile range (Q1-Q3) of the combined groups, and circles inside the box represent median values. **g**, **h** Top enriched biological processes in tumor regions with (**g**) high and (**h**) low aggressiveness. Sizes of the circles represent the number of genes in each biological process, and colors represent *P* values of enrichment. *P* values were determined using the hypergeometric test and adjusted by the Benjamini-Hochberg procedure. Source data are provided as a Source Data file.

The results presented so far have established a connection between transcriptional subtype compositions and spatial architecture with patient prognosis. To further explore spatial gene expression programs associated with prognosis, we developed a separate deep learning model that used histology images to predict prognosis (Fig.4a). The model aimed at assigning an aggressive score to each patch, where higher scores contributing to a worse prognosis. We evaluated the model’s performance through a five-fold cross-validation using data from the TCGA-GBM cohort (*n* = 693 slides from *n* = 312 patients). Additionally, we tested the model trained on the TCGA cohort on the CPTAC cohort (*n* = 227 slides from *n* = 98 patients). To assess the accuracy of the model, we derived a composite score (CS) that combined the concordance index (C-index)^41,42^ and integrated brier scores^43,44^ (“Methods”). The CS ranges from 0.0 to 1.0, with higher values indicating more accurate predictions. In the TCGA cohort, the model achieved a CS of 0.74 (SD: 0.03). Furthermore, we divided the patients into a high-risk and a low-risk group using the median predicted score. Patients in the high-risk group showed significantly worse prognosis compared to patients in the low-risk group (Supplementary Fig. 4a, log-rank test, *P* = 2.47E-07). To benchmark the model’s performance, we compared it to a baseline model, where aggressive scores were predicted by a random, untrained model with the same architecture. The CS for the trained model was significantly higher than that of the baseline model (Supplementary Fig. 4b, Mann-Whitney U test, *P* = 0.004). Similarly, in the CPTAC testing cohort, the model achieved a CS of 0.75, and patients assigned with high aggressive scores had significantly worse prognosis compared to those with low aggressive scores (Supplementary Fig. 4c, log-rank test, *P* = 0.03).

To identify survival-associated spatial gene expression programs, we next used the trained prognostic model to predict an aggressive score for each spot (*n* = 69,647) in the spatial transcriptomics cohort using the paired histology images (Fig. 4b). Our analysis revealed that the aggressive scores were significantly different between transcriptional subtypes (Fig. 4c–e). The MES-hypoxia subtype was assigned with the highest aggressive scores, followed by the reactive astrocytes, MES-like, NPC-like, and OPC-like cells (Fig. 4e). To identify genes associated with prognosis, we divided all spots using the median score of this cohort, and spots assigned with high scores were compared to those with low scores. We discovered 4,569 genes that were significantly upregulated in regions with high aggressiveness (Fig. 4f and Supplementary Data 3, log2FC > 0.25, *P* < 0.01) and 1,984 genes significantly upregulated in regions with low aggressiveness (Supplementary Data 4, log2FC > 0.25, *P* < 0.01). Gene set analysis showed genes related to high aggressiveness (Fig. 4g) were involved in the regulation of injury response (*SOD2*, *TNR, PTN*), glycoprotein metabolic process (*MT3*, *RAMP1*, *CST3*), antigen processing (*AZGP1*, *CD74*, *HLA-DRA*), response to oxidative stress (*RHOB, PON2, AQO1*), and gliogenesis (*PTPRZ1*, *GFAP*, *SOX4*). On the other hand, genes related to low aggressiveness (Fig. 4h) were associated with neuronal development, including neural nucleus development (*MBP*, *CALM1*, *CNP*), oligodendrocyte differentiation (*PLP1*, *OPALIN*, *MAG*), neurotransmitter transport (*SNAP25*, *SYT1*, *SLC17A7*) and axon development (*STMN1*, *UCHL1*, *CCK*). Some genes were known to be associated with GBM prognosis, such as *PTPRZ1*^45^ and *EGFR*^46^. However, we identified many other genes that were previously unknown, such as *SNRPD3, TPST1* and *GUCD1*. These data demonstrated that the reactive transformations of malignant cells in response to hypoxic environment and inflammatory stimuli contributed to a worse prognosis.

### Software for predictions of spatial transcriptional subtypes and aggressiveness

**Fig. 5:**
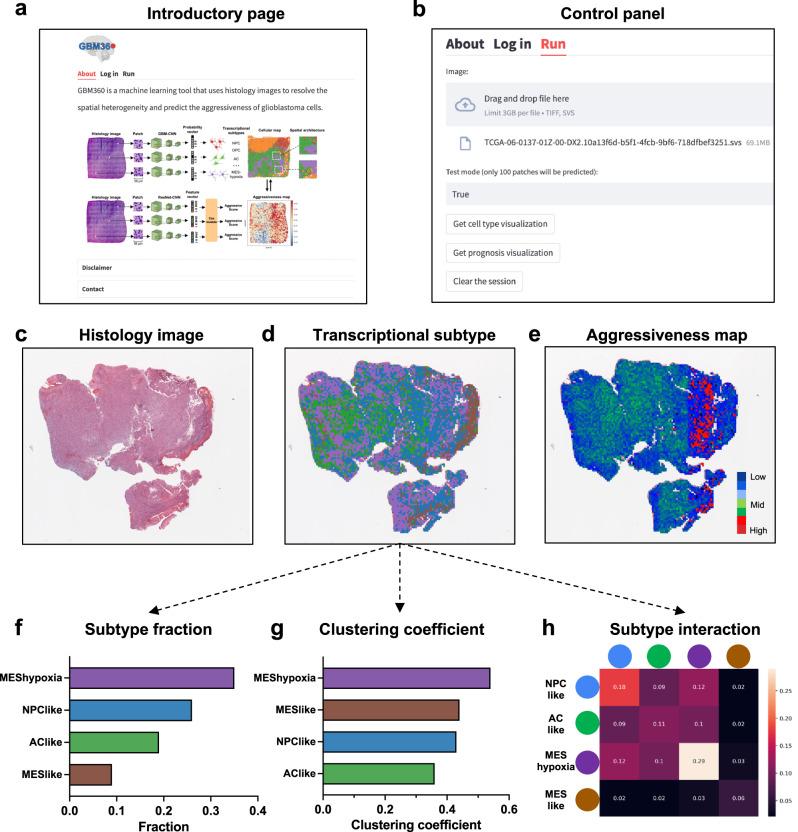
Screenshots of the GBM360 software. **a** Introductory page describing the functions of GBM360. The cell-type cartoons were created with BioRender.com. **b** Control panel for uploading histology images and configuring software settings. **c** Thumbnail of a histology image uploaded from the user. **d** Predictions and spatial visualization of the cell-type distribution. The image was colored by transcriptional subtypes. **e** Predictions and visualization of regional aggressive scores. The image was colored by the aggressive score predicted at each patch. Red indicates high aggressiveness and blue indicates low aggressiveness. **f**–**h** Statistical analysis of the transcriptional subtype distribution: (**f**) bar graph showing the transcriptional subtype fractions, (**g**) clustering coefficient for each subtype, and (**h**) two-dimensional matrix showing the frequency of interactions between any two transcriptional subtypes.

To make our trained image models accessible for future research, we developed GBM360 (https://gbm360.stanford.edu), a user-friendly software for the prediction and visualization of transcriptional subtypes and prognosis in GBM histology images (Fig. 5a). With GBM360, users can upload H&E histology images in the *svs* or *tiff* format (Fig. 5b, c). The software offers three key functionalities: (1) predicting transcriptional subtypes of GBM cells from histology images and visualizing the resulting spatial cellular maps (Figs. 5d), (2) predicting and visualizing regional aggressive scores (Figs. 5e), and (3) performing various statistical analysis for characterizing transcriptional subtype compositions, spatial cellular organization and subtype interactions (Fig. 5f, g, h).

## Discussion

The emerging spatial transcriptomic technologies have enabled transcriptomic profiling while preserving the tissue architecture. In addition, high-resolution histology images are readily available in spatial transcriptomics, providing a key opportunity for the integration of molecular characteristics and histological features. Here, we integrated data from single-cell RNA-seq, spatial transcriptomics and histology images to resolve the spatial cellular heterogeneity in GBM. The results presented have the potential to improve our understanding of how spatial cellular architecture was associated with patient prognosis.

In the past decade, deep learning-based computational approaches have demonstrated great potentials in revolutionizing tumor diagnosis. A number of deep learning algorithms, such as convolutional neural networks (CNNs), have been trained to extract intricate patterns and features from H&E histology sections. These algorithms have shown effectiveness in various aspects of tumor diagnosis, including tumor grading, subtyping, and prediction of patient outcomes^20–26^. To obtain deeper biological insights from histology images, subsequent studies have applied deep learning to predict molecular traits, such as gene expression^27^, mutations^28^, copy number alterations^29^ and hormone receptor status^30^. He et al. developed ST-Net, a CNN-based algorithm, to predict the expression of spatially variable genes in breast cancer using histology images^47^. Subsequent work by Zeng et al. applied Vision Transformer-based deep learning models to predict spatial gene expression in breast cancer^48^. Despite the importance of predicting transcription levels of individual genes, it is also critical to consider the interactions of genes within modules, where a group of functionally related genes collectively define the transcriptional states of malignant cells. In the current study, we integrated data from single-cell RNA-seq and spatial transcriptomics to identify spatially resolved gene expression programs in GBM. Our analysis based on the cNMF lead to the discovery of five meta-gene modules, including NPC-like, OPC-like, reactive astrocytes, MES-like, and MES-hypoxia. Comparative analysis of the top-scoring signatures with published gene expression modules showed that the detected cNMF modules were congruent with the existing classification of GBM cells^5,8,10,37,49^.

Despite the rapid development of the spatial technologies, their application as routine diagnostic assay is limited by their high costs and the requirement of specialized expertise. In contrast, histology images are widely available and cheaper to obtain. In this study, we tested whether the transcriptional subtypes of GBM cells could be inferred from histology images. To tackle this challenge, we developed GBM-CNN, a deep-learning model that uses histology images to predict the transcriptional subtypes of GBM cells. The model was trained and evaluated using spatial transcriptomics data and subsequently validated in external testing cohorts. Using GBM-CNN, we phenotyped over 40 million tissue spots from 920 whole-slide images across two independent cohorts, enabling the computational reconstruction of high-resolution cellular maps in 410 GBM patients. Our analysis revealed that each tumor was composed of three to five malignant transcriptional subtypes, with over 75% of the tumors consisted of all five transcriptional subtypes, highlighting the intra-tumoral cell-state heterogeneity. Integrating the predicted cellular maps with clinical data led to the discovery of survival-associated spatial cellular compositions. Notably, a higher proportion of the MES-hypoxia subtype correlated with a worse prognosis. This result is supported by recent studies showing that the hypoxic environment drives metabolic alterations of tumor cells, leading to the accumulation of genomic instabilities and epigenetic disorders, ultimately driving increased aggressiveness and therapeutic resistance^50–52^. By enabling automated detection of hypoxic regions in histology images, our model holds the potential to enhance diagnosis and facilitate personalized treatment strategies.

In addition to the composition of transcriptional subtypes, the spatial cellular organization plays a critical role in driving clonal evolution, tumor progression, and therapeutic resistance^12–15^. Through the analysis of transcriptional subtype interactions and clustering coefficient, we found that a clustering pattern of the AC-like tumor cells was associated with poor patient prognosis. Conversely, when the AC-like tumor cells were dispersed and connected to the other subtypes, the prognosis was improved. In line with this result, a recent biological study showed that the GFAP+ astrocytoma cells frequently form ultra-long membrane protrusions, known as tumor microtubes (TMs)^12^. TMs connect astrocytoma cells with each other, leading to the formation of multicellular anatomical networks^12^. In vivo studies showed that the microtube-connected cellular networks are resistant to the cytotoxic effects of radiotherapy^12^. In response to radiotherapy, microtube-connected cells are protected from cell death, while unconnected cells die in relevant numbers. In our results, the highly clustered reactive astrocytes may represent the radioprotective, microtube-connected astrocyte cellular networks. The presented results have the potential to improve our understanding of how the spatial cellular organization contributes to tumor evolution and disease progression.

To further identify survival-associated transcriptional programs, we developed a separate deep learning model that uses histology images to predict prognosis. The model was trained on the TCGA cohort and further tested in the CPTAC cohort. Applying the trained model to paired histology images of the spatial transcriptomics led to the identification of regional gene expression programs associated with prognosis. We identified both known gene expression markers for GBM prognosis, such as *PTPRZ1*^45^ and *EGFR*^46^, as well as markers that were previously uninvestigated, such as *SNRPD3, TPST1* and *GUCD1*. Overall, genes upregulated in high-aggressive regions were related to glycoprotein metabolism, antigen processing and response to axon injury. These results were congruent with our observations that the reactive transformation of malignant cells in response to metabolic and inflammatory stimuli was associated with a worse prognosis.

Limitations include the resolution for classifications of transcriptional subtypes at both the cellular and spatial dimensions. Due to the limitations of platform sensitivity, our model predicted transcriptional subtypes at a patch level rather than at a single-cell level. While our deconvolution analysis revealed that each spot was predominantly occupied by one tumor cell type, it is important to note that the tumor cells were often intermixed with immune cells, such as T cells and macrophages. Previous studies utilizing image cytometry and single-cell RNA-seq have identified the existence of distinct subsets of T cells and macrophages within the tumor microenvironment^11,53^. Notably, different immune cell subsets exhibited distinct functions in regulating tumor progression^54^. However, due to the trade-off between resolution and accuracy, we did not differentiate between different subsets of these immune cells in our deconvolution analysis. Consequently, our prognosis analysis was limited to tumor cells without considering the contribution of these immune cells. Future investigations into the interactions between tumor cells and immune cells will substantiate our understanding of how the tumor microenvironment influences disease progression and therapeutic responses.

In summary, we proposed a machine-learning framework that integrates histology images, spatial transcriptomics and patient clinical outcomes. The proposed framework offers an efficient and cost-effective approach for characterizing intra-tumoral cellular heterogeneity in GBM. Our results linked tumor compositions and the spatial cellular organization to patient prognosis. Although we demonstrated the value of our framework in GBM, it can be extended to other diseases.

## Methods

### Preprocessing of the spatial transcriptomics data

We used four publicly available spatial transcriptomics datasets comprising both tumors and normal brain tissues (Supplementary Data 1)^16,18^. All datasets were generated using the 10X Visium platform. Quality control was performed by the cell ranger pipeline and imported into AnnData objects using the Scanpy software (version 1.9). In each sample, we removed spots with less than 200 detected genes and more than 5% mitochondrial RNA. Additionally, genes detected in less than 3 spots were removed. Given the potential presence of batch effects in spatial transcriptomics data, we performed normalization and variance stabilization across different samples using regularized negative binomial regression^55^. We regressed out percentages of mitochondria-expressed genes per spot and effects from cell cycles. This approach allowed us to remove the influence of technical variances from downstream analyses while preserving biological heterogeneity.

### CNA inference and prediction of tumor cell content

We used the InferCNV method^56^ to estimate copy number alterations (CNAs) of each spot from GBM tissues, where the transcriptomes of a separate cohort of normal brains (*n* = 6 tissues from *n* = 3 patients) were used as a ref. ^57^. We calculated an average gene expression value over a chromosomal window (default = 100 genes) across each analyzed gene/chromosomal region in GBM tissues and compared the value to its counterpart in normal brains. The output from InferCNV was a two-dimensional matrix indicating the CNA score of each chromosomal window in each spot. We then rescaled the CNA matrix, such that at each chromosomal window, the CNA score of normal brains ranged between 0.98 and 1.02, with an average score of 1.00. Compared to normal brains, tumor tissues exhibited a broad CNA across genome, such as gain of Chr 7 and loss of Chr 10. Then we selected a signature CNA event in each tumor that shared across all its spots. To define tumor signature CNAs, we required the average CNA score > 1.05 if the signature was a chromosomal gain or <0.95 if the signature was a chromosomal loss. The tumor cell content $C$ for a given spot $i$ was defined as ${C}_{i}=\frac{\left[{A}_{{CNVi}}-1\right]}{\left[{{\max }}\left({A}_{{CNV}}\right)-1\right]}$ if the signature was chromosomal gain and ${C}_{i}=\frac{\left[1-\,{A}_{{CNVi}}\right]}{\left[1-{{\min }}\left({A}_{{CNV}}\right)\right]}$ if the signature was chromosomal loss. At least three tumor signature events were used in each tumor, and the average results were calculated to ensure robust and unbiased estimation. Spots with $C$ > 0.2 were defined as malignant spots and retained for downstream analysis.

### Consensus non-negative matrix factorization (cNMF)

To identify gene expression programs (i.e., meta-gene modules) that govern the transcriptional phenotypes of malignant cells, we used the cNMF algorithm (version 1.3.4)^32^. We aimed to generate an unbiased classification of transcriptional subtypes across the patients, where we didn’t assume that a transcriptional subtype can always be found in every patient and one tumor may not include all transcriptional subtypes. Therefore, we ran the cNMF using transcriptomes pulled from all patients. Given the high levels of inter-patient heterogeneity, it is possible that some transcriptional subtypes were present in only a subset of the patients but missing in other patients. Non-negative matrix factorization was run 200 times for *k* clusters, where *k* ranged from 2 to 15. The optimal *k* value was selected by finding the most stable clustering solution, in which the maximum clustering stability and lowest error rate were found.

### Gene set analysis

Gene set analysis was performed with the *clusterProfiler* R package (version 4.2.1)^58^. We selected the top 100 scoring genes of each meta-gene module, and hypergeometric testing was used to identify enriched biological processes using the gene ontology (GO). To determine significant biological processes, we set the *P* value to 0.05 and Q value to 0.20.

### Spatially weighted correlation analysis

To correlate the cNMF modules defined from our study to published gene expression modules, we performed spatially weighted correlation analysis. We first scored each module in each spot using the *AddModuleScore* function from the Seurat R package (version 4.3.0)^59^. We then correlated any two set of modules using geographically-weighted regression using the GWmodel package (version 2.2)^60^.

### Preprocessing and integration of single-cell RNA-seq data

To include both tumor cells and immune cells as references for deconvolution analysis, we integrated three single-cell RNA-seq datasets: GSE131928, GSE163108, and GSE84465 (Supplementary Data 1)^5,11,37^. GSE131928 predominately contains tumor cells, while GSE163108 predominately contains immune cells. GSE84465 contains both tumor and immune cell types. We performed preprocessing and batch effect normalization following the same procedures as outlined in the previous section. Specifically, we used the SCTransform algorithm to regress out percentages of mitochondria-expressed genes per cell and cell-cycle effects^55^. To stratify tumor cells into different transcriptional subtypes, we used the gene expression modules derived from GSE131928, which comprised GBM cells from 28 patients. Module scores were calculated for each tumor cell using the AddModuleScore function from the Seurat R package (version 4.3.0)^59^, and the cell type was assigned based on the module with the highest score. For the subsequent deconvolution analysis, we randomly selected 20% of cells from each transcriptional subtype as the reference. In the case of immune cells, we randomly sampled 200 CD4 T cells and 200 CD8 T cells from GSE163108 and combined them with the immune cells from the other two datasets. Considering a balanced trade-off between resolution and accuracy of deconvolution, we merged CD4 and CD8 T cells into a single cell-type label. To generate a UMAP visualization of the integrated single-cell dataset, we normalized the total counts across all genes to ensure that every cell had the same total counts after normalization. The number of neighbors was set to 15, and the neighborhood graph was embedded into two dimensions using UMAP, and visualization was generated using the *sc.pl.umap()* function of the Scanpy software (version 1.9).

### Align single-cell RNA-seq data to spatial transcriptomics

To deconvolute the spots obtained from spatial transcriptomics, we first determined the number of cells present in each spot. For this purpose, we performed nuclei segmentation on H&E-stained histology images using the StarDist algorithm (version 0.8.3, https://github.com/stardist/stardist)^61^. The accuracy of segmentation was confirmed through visual inspection. To estimate the fractions of different cell types within each spot, we constructed a reference dataset using the single-cell RNA-seq data, as described in the section above. To map the single cells to spots, we used the Tangram algorithm (version 1.0.4)^62^. In this process, we selected the top 200 differentially expressed genes between different single-cell clusters as training genes. To validate the deconvolution results, we utilized another set of 200 genes as testing genes, and we calculated the alignment score for each gene.

### Image processing and data augmentation

#### Spatial transcriptomics cohort

For the training and internal validation of GBM-CNN, we used data from the spatial transcriptomics cohorts. All images were taken at 20x magnification. We enhanced the brightness and contrast of each image by 1.5 times, and the quality of the images were confirmed by visual inspection. Given the variation in H&E staining colors across different samples, we performed stain normalization using StainTools (version 2.1.2, https://github.com/Peter554/StainTools). For this purpose, we randomly selected 20 histology images from the TCGA-GBM cohort as references, and we normalized the stain colors of each image of the spatial transcriptomics to match those of each reference. The average image features derived from all references were used for further analysis.

To extract patches, we centered each patch at the spatial coordinate of its corresponding gene expression spot. Each patch had an edge length of 56 μm, which was approximately equal to the diameter (55 μm) of a single gene expression spot. The Pillow image Library (Version 9.2.0) was utilized for patch extraction, and the extracted patches were resized to 224 × 224 pixels. During the training phase of GBM-CNN, we performed image augmentation, including random horizontal and vertical flipping in 50% of the time, as well as random adjustments of brightness (factor = 0.25), contrast (factor = 0.25), and saturation (factor = 0.25).

#### TCGA, CPTAC and IvyGAP cohorts

Histology images of the TCGA-GBM cohort were obtained from the Genomic Data Commons (GDC) portal using a Data Transfer Tool Client (https://gdc.cancer.gov/access-data/gdc-data-transfer-tool). Histology images of the CPTAC-GBM cohort were download from the Cancer Image Archive (https://www.cancerimagingarchive.net/collections). The accession URLs were listed in Supplementary Data 1. Histology images of the IvyGap cohort were downloaded from the Ivy Glioblastoma Atlas Project (https://glioblastoma.alleninstitute.org) using the “Requests” HTTP library (version 2.31) in Python.

We used whole-slide images (WSIs) of formalin-fixed, paraffin-embedded (FFPE) diagnostic slides captured at 20x magnification, corresponding to a pixel resolution of 0.5 $\mu m$/pixel. To separate tissue sections (foreground) from the white background, we applied an Otsu segmentation mask to each WSI. Given the observed variation in H&E stain colors between the TCGA and CPTAC cohorts, we performed stain normalization using StainTools (version 2.1.2, https://github.com/Peter554/StainTools). We randomly selected 20 histology images from the TCGA cohort as references and normalized the color of each CPTAC image to match the stain colors of each reference. The average image features derived from all reference images were then used for subsequent analysis.

To extract patches, we used the OpenSlide library (Python API, version 1.2.0)^63^. Each patch had an edge length of 56$\mu m$ (112 $\times$ 112 pixels), which was consistent with the patch size extracted from the spatial transcriptomics cohort. Each patch was then subsequently converted to 224 $\times$ 224 pixels as input to the model. If a patient had multiple slides, we included all of them for the presented analysis.

### Architecture of GBM-CNN and training algorithm

All deep learning models were implemented with the PyTorch library (version 2.0). To extract histology features from each 224 $\times$ 224 pixel patch, we used a ResNet-50 module^64^. Each patch was mapped to a feature vector of size 2048. To enhance model performance, we adopted a Transfer Learning approach in which the ResNet-50 module was initialized with weights pre-trained on ImageNet^65^. During the training phase, we selected the last two layers of the ResNet blocks to fine-tune while freezing the other ResNet blocks. The feature vector was then converted to a probability vector through a fully connected layer. To optimize model weights, we used ADAM^66^ as the optimizer and the cross-entropy as the loss function. The training parameters were selected empirically, with the mini-batch size set to 64, the learning rate set to 5e-4, and the weight decay set to 1e-5. The model was evaluated using leave-one-out cross-validation (LOOCV). In each iteration, the model was trained for five epochs on the training samples and validated using the sample left out for validation. Following the LOOCV, we trained a final model using data from all samples (n = 23) in the cohort. This final model was used to predict transcriptional subtypes in images obtained from external cohorts (i.e., TCGA, CPTAC, IvyGAP).

### Infer cell-type proportions from bulk RNA-seq data

To estimate the fraction of each transcriptional subtype from the bulk RNA-seq, we used the CIBERSORTx algorithm^67^. To construct a signature gene expression matrix, we randomly selected 100 cells from each transcriptional subtype from our integrated single-cell RNA-seq dataset (refer to the “Preprocessing and integration of single-cell RNA-seq data” section for details). For the bulk RNA-seq data, we obtained the raw gene expression counts of the TCGA-GBM cohort from the UCSC Xena browser^68^. We performed the deconvolution analysis using the default parameters of CIBERSORTx, which provided us with the estimated proportions of each transcriptional subtype in each patient.

To establish a correlation between the fraction of transcriptional subtypes obtained from the deconvolution analysis of bulk RNA-seq and those predicted from histology images, we focused exclusively on frozen tissues from the TCGA cohort (n = 338 slides from n = 166 patients). These frozen tissue slides were derived from the same resected regions as the tissues used for bulk RNA-seq analysis. For all other analysis, the FFPE tissues were used as stated in the previous section.

### Extraction of histological features

To assess whether the transcriptional subtypes correlated with any raw histological features, we extracted (1) the color values from each image channel, including their mean and quantiles, (2) histogram features and (3) texture features. The histogram features quantified histogram counts of color channel values, while texture features characterized the different combinations of distance and angle between pixels. The combined feature dimension was 105 $\times$ 1. The feature extraction was performed with the Squidpy Python library (version 1.2.2)^69^.

### Image-based aggressive score predictions

#### Model architecture and training algorithm

Similar to GBM-CNN, we used a ResNet-50 module^64^ to extract histopathological features from patches, and each patch was converted into a one-dimensional feature vector $Z$, where the size of $Z$ was 2,048. The feature vector $Z$ was then mapped to an aggressive score through a fully connected layer implementing the Cox loss.

The goal of survival prediction is to predict the likelihood that the patient will survive until time $t$ given patient features $Z$. We used the Cox proportional hazards model to predict patient survival based on the feature vector, where the hazard function was $\lambda \left(t|Z \right)$ = ${\lambda }_{0}(t){{\exp }}(Z\bullet \beta )$. The ${\lambda }_{0}\left(t\right)$ was the baseline hazard function, and $\beta$ was the corresponding coefficient weight implemented in the fully connected layer. The Cox model was able to include censored data in case where the death time of some patients were unknown (either they were still alive, or we lost the track of their information at a certain time point). Let ${Z}_{i}$ be the features of the patient $i$, ${Y}_{i}$ be the survival time, and ${C}_{i}$ be the censor indicator, we have ${C}_{i}\,={\delta }_{{event\; i}={death}}$. The negative log-likelihood to minimize (or Cox loss) was

1 $${{{{{\mathcal{L}}}}}}\left(\beta \right)=-\mathop{\sum}\limits_{i{{{{{\rm{|}}}}}}{C}_{i}=1}\bigg({Z}_{i}\beta -{{\log }}\bigg(\mathop{\sum}\limits_{{j{{{{{\rm{|}}}}}}Y}_{j}\ge {Y}_{i}}{e}^{{Z}_{j}\beta }\bigg)\bigg).$$

We adapted this loss to a deep learning framework. Since ${Z}_{i}$ was the extracted features of patient $i$, ${Z}_{i}\beta$ can be represented by ${f}_{\theta }\left({X}_{i}\right)$ in the neural network setting, where ${X}_{i}$ was the input predictor of patient $i$, $f$ denoted a nonlinear mapping the neural network learns to first extract patient features from the predictor and to finally predict patient risk, and $\theta$ denoted the model parameters including the weights and biases of each neural network layer. Our objective to minimize was

2 $${{{{{\mathscr{L}}}}}}\left(\theta \right)=-\mathop{\sum}\limits_{i{{{{{\rm{|}}}}}}{C}_{i}=1}\bigg(\,{f}_{\theta }\left({X}_{i}\right)-{{\log }}\bigg(\mathop{\sum}\limits_{{j{{{{{\rm{|}}}}}}Y}_{j}\ge {Y}_{i}}{e}^{{\,f}_{\theta }\left({X}_{j}\right)}\bigg)\bigg).$$

In practice, we can’t compute the sum $\mathop{\sum}\limits_{{j{{{{{\rm{|}}}}}}Y}_{j}\ge {Y}_{i}}{e}^{{\,f}_{\theta }\left({X}_{j}\right)}$ over all patients. We adopt a batch sampling strategy and compute this sum with patients of each batch.

For training the model, we initialized the ResNet50 module with the weights of a model pretrained on ImageNet^65^, and we selected the last two layers of the ResNet blocks to fine-tune while freezing the other blocks. The training is patch-based, and the model aimed at predicting aggressive scores for patches. In testing, we averaged aggressive scores of all patches from a patient to get the final aggressive score. Since training a model using all patches from a WSI could be computationally expensive, we randomly selected 200 patches from each patient. This number was determined based on a balanced consideration between performance and computational time after testing a range of different number of patches. We used ADAM^66^ as the optimizer with cross-entropy as the loss function to optimize the model weights. The mini-batch size was set to 128 and the learning rate was 5e-4.

#### Evaluation of algorithm

We initially considered two standard evaluation metrics for testing the performance of a prognosis prediction model: (1) the concordance index (C-index)^41,42^ and (2) the integrated Brier score (IBS)^43^. The C-index is a performance measure that evaluates how well the predicted aggressive score ranks patients according to their actual survival time. It was calculated by dividing the number of all pairs of subjects whose predicted risks are correctly ordered, by the number of admissible pairs of subjects. A pair is considered admissible if neither event in the pair is censored, or the earlier time in the pair is not censored. A value of 1.0 indicates perfect prediction where all the pairs are correctly ordered, and a value of 0.5 indicates random prediction. The Brier score was calculated by the squared differences between observed survival status and the predicted survival probability at a given time point. The IBS provided an overall evaluation of the model performance at all available times. In contrary to the C-index, an IBS closing to 0.0 indicates good prediction, while an IBS closing to 1.0 indicates poor prediction.

Since GBM is a highly aggressive cancer type, and patient survival time is relatively short and homogenous, IBS is a more relevant evaluation compared to C-index^44^. Therefore, we derived a composite score (CS) that integrated both IBS and C-index:

3 $${{{{{\rm{CS}}}}}}=\frac{{{{{{\rm{C}}}}}}-{{{{{\rm{index}}}}}}+\left(1-{{{{{\rm{IBS}}}}}}\right)}{2}\,.$$

The C-index was calculated using the “lifelines” package (version 0.27.4) in Python, and the IBS was calculated using the “survcomp” package (version 3.16) in R.

### Spatial statistical analysis

#### Spatial neighborhood graph

To characterize the spatial cellular organization, we first built a spatial neighborhood graph on each WSI, where nodes are patches and edges are direct interactions between the patches. We used spatial coordinates of each patch to identify neighbors among them. We defined the neighbors of a patch as those patches that were located within a two-patch distance (maximum of 24 patches from 5 × 5 patches). The class phenotype at each patch is the predicted transcriptional subtype. The neighborhood graph can be denoted as $G=\left(V,{E}\right)$, where $V$ represents vertices (nodes) and $E$ represents edges between the vertices. The neighborhood graph was implemented using the Python NetworkX libarary^70^.

#### Clustering coefficient

The clustering coefficient measures how well nodes of a specific class tend to cluster together. It is defined as the ratio of the number of interactions ($I$) between the class members to the number of all interactions that includes that class member:

4 $${{Cluster}}_{\left(m\right)}=\frac{I\left(M,\,M\right)}{I\left(M,\,M\right)+I\left(M,\,K\right)}\in \left[0,\,1\right]$$

where $K$ represents any class that is not class $M$.

#### Interaction matrix

Interaction matrix represents the number of edges between any two malignant cell types ($M$ and $K$) divided by the total number of edges between all malignant cell types in the graph:

5 $${{Interaction}}_{\left(m,k\right)}=\frac{I\left(M,\,K\right)}{\mathop{\sum}\limits_{i,\,j\,\in \,V} \, I\left(i,\,j\right)}.$$

### Generation of abstractive networks

To enhance the visualization of spatial cellular interactions, we generated abstractive networks based on the predicted cellular maps. We first selected a region of interest (e.g., region with clusters of the AC-like subtype) from the spatial neighborhood graph of the image. Then, for each node in the selected region, we inspected its neighboring nodes. If over 70% of the neighboring nodes belong to the same transcription subtype as the target node, we aggregated all nodes in this neighborhood into one node. This process was repeated for every node in the selected region until converging. The resulting abstractive networks were visualized using the neworkD3 library (version 0.4).

### Survival analysis

Molecular characteristics and clinical endpoints of GBM patients were obtained from published studies of the TCGA^71,72^ and CPTAC^40^ cohorts. Multivariate Cox regression analysis and the log rank test were performed using the lifelines package (version 0.27.4) in Python (version 3.9)^73^. In Cox regression analysis, we included gender, age, tumor size and IDH subtype as covariates. The *P* values were adjusted for multiple testing using the Benjamini-Hochberg method.

### Reporting summary

Further information on research design is available in the Nature Portfolio Reporting Summary linked to this article.

## Supplementary information

Supplementary Information

Peer Review File

Description of Additional Supplementary Files

Supplementary Data 1

Supplementary Data 2

Supplementary Data 3

Supplementary Data 4

Reporting Summary

## Data Availability

All datasets analyzed in the current study, including spatial transcriptomics, single-cell RNA-seq and histology images, are publicly accessible, with the accession URLs listed in Supplementary Data 1. The single-cell RNA-seq data were obtained from the GEO database under the following accession numbers: GSE131928^5^, GSE163108^11^, GSE84465^37^. The publicly available spatial transcriptomics data were acquired using the following accession URLs: (1) Datadryad [10.5061/dryad.h70rxwdmj]^16^; (2) Figshare [10.6084/m9.figshare.20653908.v3]^18^ (3) 10X Genomics [https://www.10xgenomics.com/resources/datasets/human-glioblastoma-whole-transcriptome-analysis-1-standard-1-2-0]; (4) LIBD [http://research.libd.org/spatialLIBD]^31^. The in-situ RNA hybridization data were obtained from the Ivy Glioblastoma Atlas Project using the accession URL [https://glioblastoma.alleninstitute.org]^38^. The publicly available histology images of the TCGA-GBM cohort were downloaded from the GDC data portal [https://portal.gdc.cancer.gov/projects/TCGA-GBM], and the bulk RNA-seq data were obtained from the UCSC Xena browser [https://gdc-hub.s3.us-east-1.amazonaws.com/download/TCGA-GBM.htseq_counts.tsv.gz]^39^. The publicly available histology images of the CPTAC-GBM cohort were downloaded from the Cancer Image Archive with the accession URL [https://www.cancerimagingarchive.net/collections], and the publicly available clinical data were obtained from the GDC data portal [https://portal.gdc.cancer.gov/projects/CPTAC-3]^40^. The remaining data are available within the Article, Supplementary Information or Source Data file. Source data are provided with this paper.

## Source data

Source Data

## Code availability

The source codes of the GBM360 software used to perform the analyses presented in this manuscript are available on GitHub at https://github.com/gevaertlab/GBM360 and Zenodo at 10.5281/zenodo.8051305^74^.
