# Supplementary material for: Spatial cellular architecture predicts prognosis in glioblastoma
Source: Nat Commun. 2023 Jul 11;14:4122. doi: 10.1038/s41467-023-39933-0 (PMC10336135; doi:10.1038/s41467-023-39933-0)
Supplement: Supplementary file 1 — Supplementary Information [file 41467_2023_39933_MOESM1_ESM.pdf]

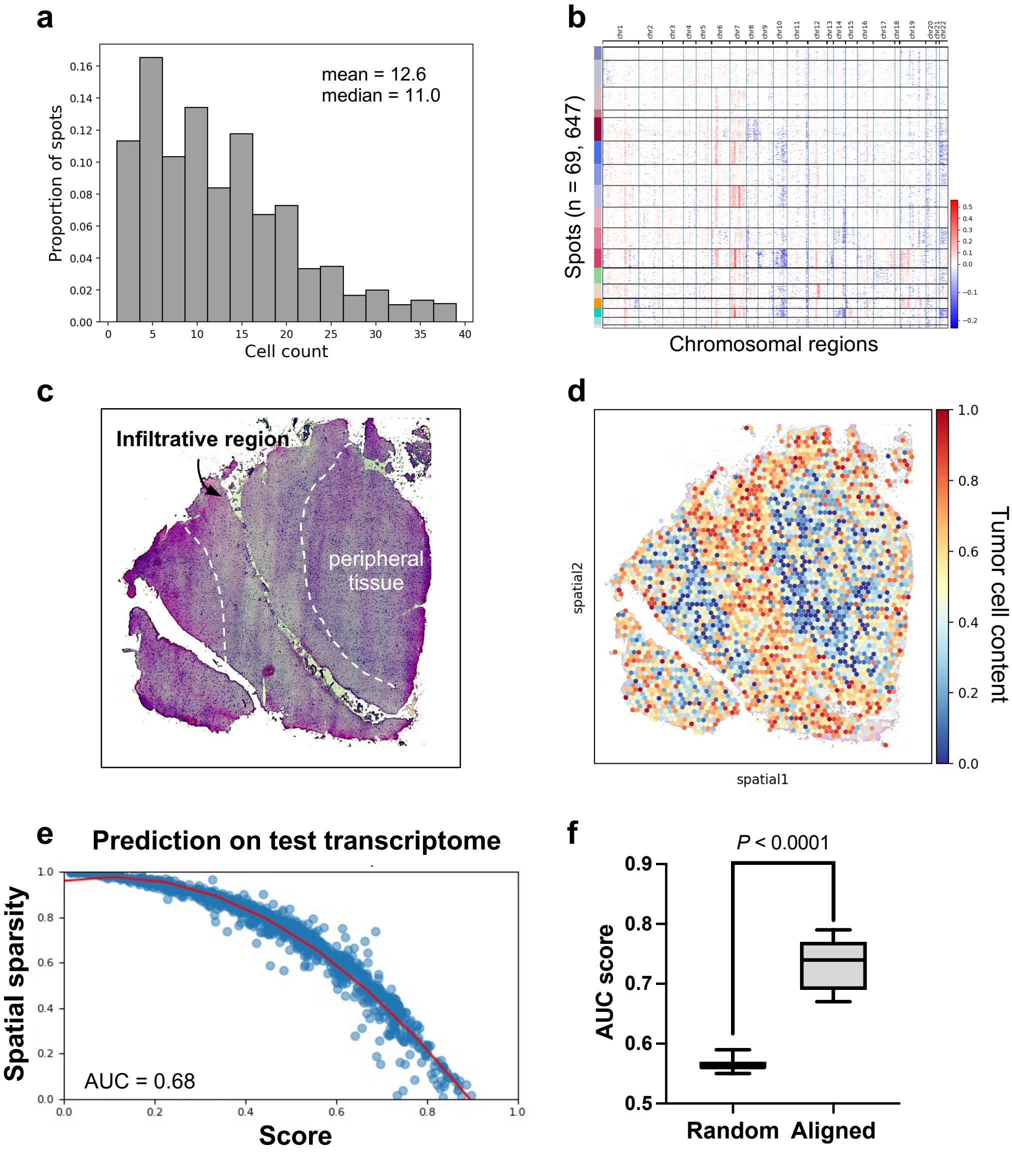

Supplementary Fig.1 (a) Cell count in each spot (total n = 75,625 spots) quantified from nuclei segmentation using histology images. **(b)** Heatmap showing inferred CNA scores across different chromosomal regions (x-axis) in malignant spots (y-axis). Spots were grouped by CNA profiles. **(c)** The histology image and **(d)** predicted tumor cell content in an infiltrative tumor. **(e-f)** Validation of the spot deconvolution: (e) Prediction score of the test genes (n = 200) in a tumor sample. Each dot represents a gene, the x-axis indicates the score, and the y-axis indicates the sparsity of that gene in the spatial data. It is expected that the prediction score drops with increase in sparsity; (f) Area under the curve (AUC) of test scores from all samples (n = 23) using the trained Tangram model (aligned) versus a baseline model where the single cells were randomly seeded to spatial data (random). Box plots represent the interquartile range (Q1-Q3) and the median value of each group, and the whiskers represent minimum and maximum values. The *P* value was determined using a two-sided Mann-Whitney U test (*P* = 3.02E-05). Source data are provided as a Source Data file.

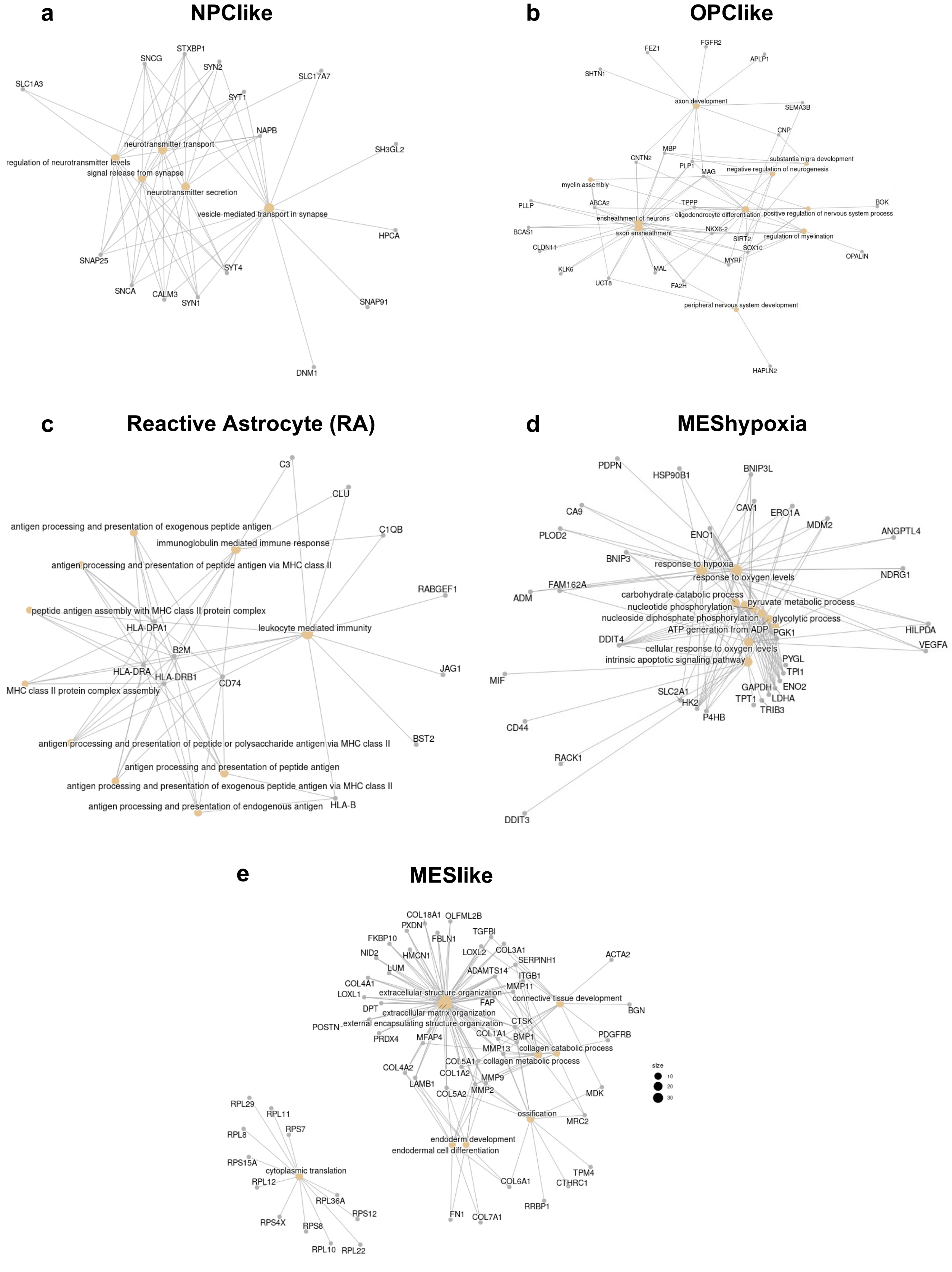

Supplementary Fig.2 (a-e) Network visualization of the enriched biological processes of each cNMF meta-module: (a) NPC-like, (b) OPC-like, (c) reactive astrocytes (RA), (d) MES-hypoxia and (e) MES-like. Nodes colored yellow represented biological processes, and grey represented corresponding gene signatures.

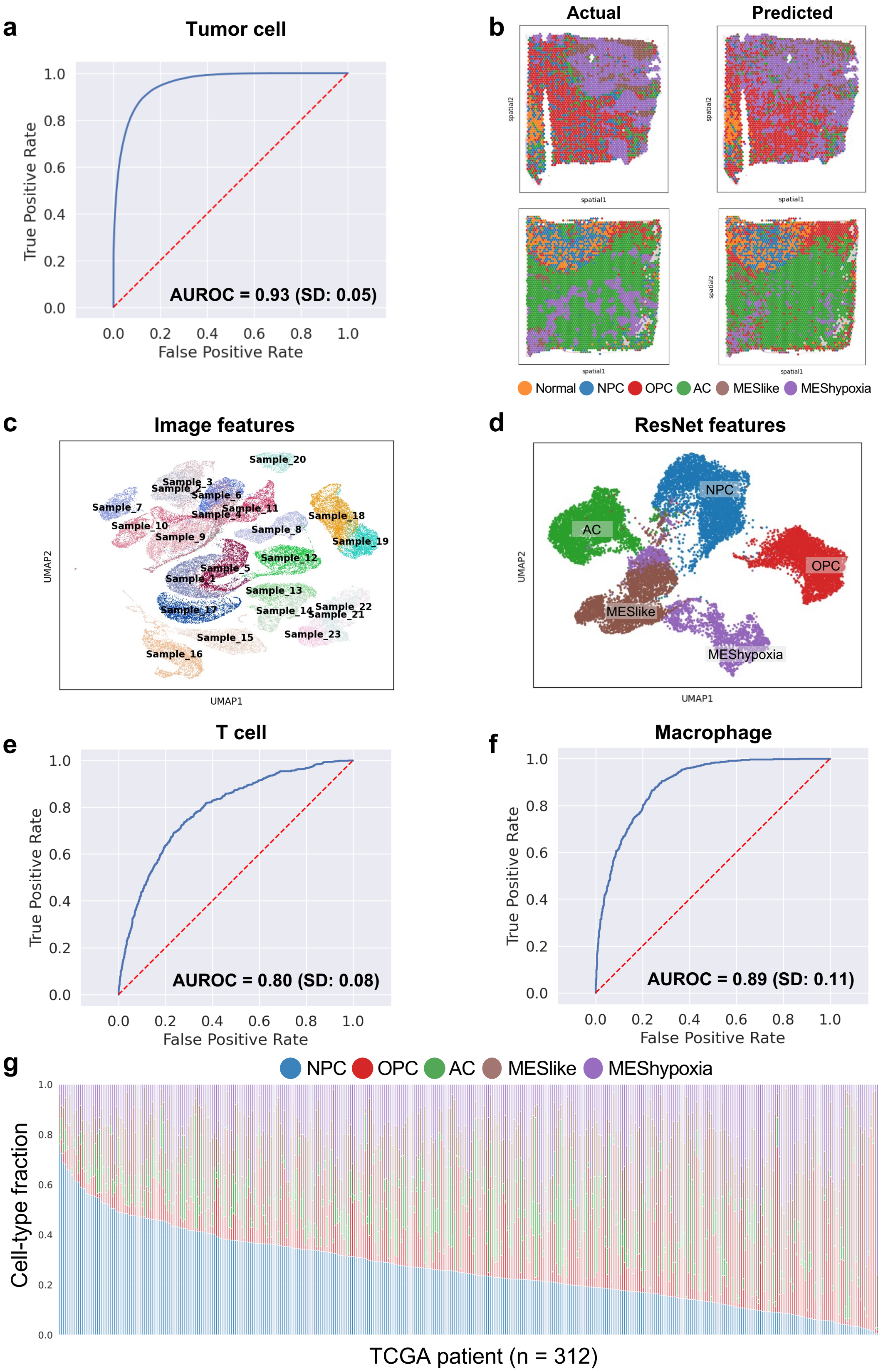

Supplementary Fig.3 (a) ROC curve showing the classification performance of GBM-CNN in predicting the dominant tumor cell type in malignant spots (n = 69, 647; n = 23 samples). Micro-average AUROC = 0.93 (SD: 0.05). **(b)** Spatial visualization of the actual and predicted distributions of dominant tumor cell types in two samples from the spatial transcriptomics cohort. **(c)** UMAP visualization of spots clustered based on raw image features. Each dot represents a spot, and the spot was colored by the originating samples (n = 23). **(d)** UMAP visualization of spots clustered based on the ResNet features from GBM-CNN. Each dot represents a spot, and the spot was colored by the dominant tumor cell type. **(e-f)** ROC curves showing the performance of GBM-CNN in predicting the presence of (e) T cell and (f) macrophage in malignant spots (n = 69, 647; n = 23 samples). **(g)** Stacked bar graph showing the distribution of malignant cell types by fractions (y-axis) of the TCGA patients (x-axis, n = 312). If a patient had more than one slide, the average fraction was shown. ROC: receiver operating characteristic curve; AUROC: area under the receiver operating characteristic curve; SD: standard deviation.

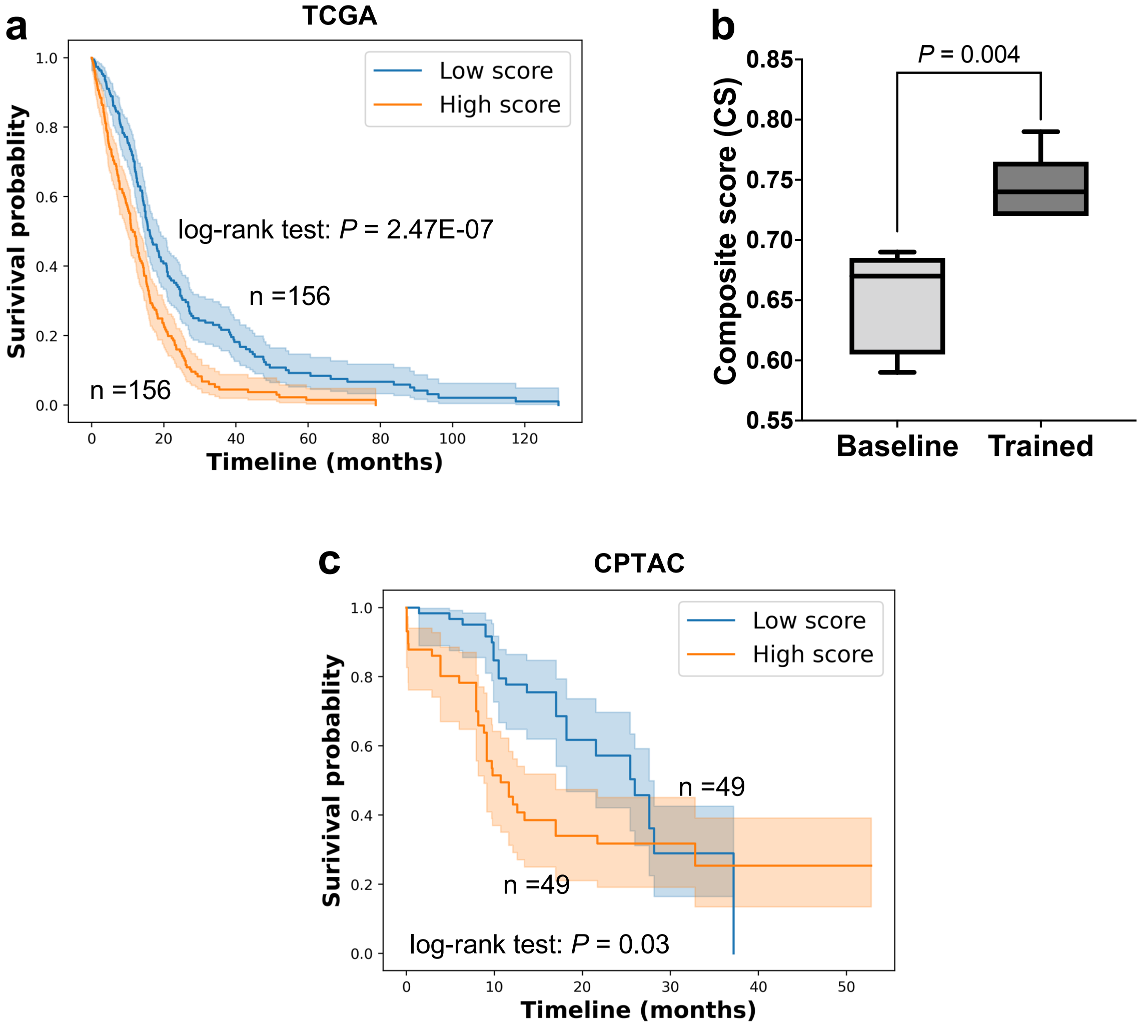

Supplementary Fig.4 (a) Kaplan-Meier survival curves for patients assigned with high and low aggressive scores in the TCGA validation cohorts (n = 312 patients). Patients were ranked by the predicted aggressive scores and were cut off by the median score. Error bands represent confidence intervals for the estimated survival probabilities, and the ground truth survival curves were compared with the log-rank test (*P* = 2.47E-07). **(b)** Composite scores from the five-fold cross-validations showing the performance of our image model (n = 312 patients) versus a random, baseline model (n = 312 patients). Box plots represent the interquartile ranges (Q1-Q3) and median scores, and whiskers represent minimum and maximum scores. The *P* value was determined using a two-sided Mann-Whitney U test. **(c)** Kaplan-Meier survival curves for patients assigned with high and low aggressive scores in the CPTAC cohort (n = 98 patients). Patients were ranked by the predicted aggressive scores and were cut off by the median score. Error bands represent confidence intervals for the estimated survival probabilities, and the ground truth survival curves were compared with the log-rank test (*P* = 0.03). Source data are provided as a Source Data file.
