# Supplementary material for: Spatial cellular architecture predicts prognosis in glioblastoma
Source: Nat Commun. 2023 Jul 11;14:4122. doi: 10.1038/s41467-023-39933-0 (PMC10336135; doi:10.1038/s41467-023-39933-0)
Supplement: Supplementary file 3 — Description of Additional Supplementary Files [file 41467_2023_39933_MOESM3_ESM.pdf]

Description of Additional Supplementary Files

File Name: Supplementary Data 1
Description: Data resources

File Name: Supplementary Data 2
Description: Top scoring genes of the cNMF modules

File Name: Supplementary Data 3
Description: Genes upregulated in high-aggressive tumor regions. *P* values were determined using the two-sided Mann-Whitney U test and adjusted for multiple comparison using the Benjamini-Hochberg procedure.

File Name: Supplementary Data 4
Description: Genes upregulated in low-aggressive tumor regions. *P* values were determined using the two-sided Mann-Whitney U test and adjusted for multiple comparison using the Benjamini-Hochberg procedure.
