# Supplementary material for: Spatial cellular architecture predicts prognosis in glioblastoma
Source: Nat Commun. 2023 Jul 11;14:4122. doi: 10.1038/s41467-023-39933-0 (PMC10336135; doi:10.1038/s41467-023-39933-0)
Supplement: Supplementary file 8 — Reporting Summary [file 41467_2023_39933_MOESM8_ESM.pdf]

nature portfolio

Corresponding author(s): Olivier Gevaert

Last updated by author(s): 2023/06/17

Reporting Summary

Nature Portfolio wishes to improve the reproducibility of the work that we publish. This form provides structure for consistency and transparency in reporting. For further information on Nature Portfolio policies, see our Editorial Policies and the Editorial Policy Checklist.

Statistics

For all statistical analyses, confirm that the following items are present in the figure legend, table legend, main text, or Methods section.

n/a	Confirmed	
☐	☒	The exact sample size (*n*) for each experimental group/condition, given as a discrete number and unit of measurement
☐	☒	A statement on whether measurements were taken from distinct samples or whether the same sample was measured repeatedly
☐	☒	The statistical test(s) used AND whether they are one- or two-sided<br>*Only common tests should be described solely by name; describe more complex techniques in the Methods section.*
☐	☒	A description of all covariates tested
☐	☒	A description of any assumptions or corrections, such as tests of normality and adjustment for multiple comparisons
☐	☒	A full description of the statistical parameters including central tendency (e.g. means) or other basic estimates (e.g. regression coefficient) AND variation (e.g. standard deviation) or associated estimates of uncertainty (e.g. confidence intervals)
☐	☒	For null hypothesis testing, the test statistic (e.g. *F*, *t*, *r*) with confidence intervals, effect sizes, degrees of freedom and *P* value noted<br>*Give P values as exact values whenever suitable.*
☒	☐	For Bayesian analysis, information on the choice of priors and Markov chain Monte Carlo settings
☐	☒	For hierarchical and complex designs, identification of the appropriate level for tests and full reporting of outcomes
☐	☒	Estimates of effect sizes (e.g. Cohen's *d*, Pearson's *r*), indicating how they were calculated

Our web collection on statistics for biologists contains articles on many of the points above.

Software and code

Policy information about availability of computer code

Data collection	Histology images of the TCGA and CPTAC GBM cohorts were retrieved from the Genomic Data Commons (GDC) portal using a Data Transfer Tool Client (https://gdc.cancer.gov/access-data/gdc-data-transfer-tool). Bulk RNA-seq data were obtained from the UCSC Xena browser. Image data from the IvyGap cohort were downloaded from the Ivy Glioblastoma Atlas Project (https://glioblastoma.alleninstitute.org) using the "Requests" HTTP library (version 2.31) in Python.
Data analysis	Our custom codes for data analysis are publicly available on a GitHub repository (https://github.com/gevaertlab/GBM360). Other software tools were described in the "Methods" section, including the Scanpy software (version 1.9), InferCNV library (verson 0.4.1), cNMF (version 1.3.4), clusterProfiler R (version 4.2.1), survcomp" R package (version 3.16), "lifelines" Python package (version 0.27.4), CIBERSORTx algorithm (Newman et al., Nat Biotech, 2019), Squidpy (v 1.2.2), StainTools (version 2.1.2), Seurat R package (version 4.3.0), GWmodel R package (version 2.2), StarDist algorithm (version 0.8.3, https://github.com/stardist/stardist), Tangram algorithm (version 1.0.4), neworkD3 library (version 0.4), PyTorch library (version 2.0), Pillow image Library (Version 9.2.0)

For manuscripts utilizing custom algorithms or software that are central to the research but not yet described in published literature, software must be made available to editors and reviewers. We strongly encourage code deposition in a community repository (e.g. GitHub). See the Nature Portfolio guidelines for submitting code & software for further information.

Data

Policy information about availability of data

All manuscripts must include a data availability statement. This statement should provide the following information, where applicable:

- Accession codes, unique identifiers, or web links for publicly available datasets
- A description of any restrictions on data availability
- For clinical datasets or third party data, please ensure that the statement adheres to our policy

The single-cell RNA-seq publicly available data used in this study are available in the GEO database under the following accession numbers: GSE131928 [https://www.ncbi.nlm.nih.gov/geo/query/acc.cgi?acc=GSE131928] [Neftel et al. 2019], GSE163108 [https://www.ncbi.nlm.nih.gov/geo/query/acc.cgi?acc=GSE163108] [Mathewson et al. 2021], GSE84465 [https://www.ncbi.nlm.nih.gov/geo/query/acc.cgi?acc=GSE84465] [Darmanis et al., 2017]. The publicly available spatial transcriptomics data were acquired using the following accession URLs: (1) Datadryad [https://doi.org/10.5061/dryad.h70rxwdmj] [Ravi et al. 2022]; (2) Figshare [https://doi.org/10.6084/m9.figshare.20653908.v3] [Ren et al. 2023]; (3) 10X Genomics [https://www.10xgenomics.com/resources/datasets/human-glioblastoma-whole-transcriptome-analysis-1-standard-1-2-0]; (4) LIBD [http://research.libd.org/spatialLIBD][Maynard et al. 2021]. The in-situ RNA hybridization data were obtained from the Ivy Glioblastoma Atlas Project using the accession URL [https://glioblastoma.alleninstitute.org]. The publicly available histology images of the TCGA-GBM cohort were downloaded from the GDC data portal [https://portal.gdc.cancer.gov/projects/TCGA-GBM] [Brennan et al. 2014]. The bulk RNA-seq data of the TCGA-GBM cohort were obtained from the UCSC Xena browser under the accession URL [https://gdc-hub.s3.us-east-1.amazonaws.com/download/TCGA-GBM.htseq_counts.tsv.gz]. The publicly available histology images of the CPTAC-GBM cohort were downloaded from the Cancer Image Archive [https://www.cancerimagingarchive.net/collections], and the publicly available clinical data were obtained from the GDC data portal [https://portal.gdc.cancer.gov/projects/CPTAC-3] [Wang et al. 2021]. The remaining data are available within the Article, Supplementary Information or Source Data file.

Human research participants

Policy information about studies involving human research participants and Sex and Gender in Research.

Reporting on sex and gender	This study used public datasets. All studies include both male and female participants, in which the selection criteria were described in the published studies. No further selection based on sex and gender was made in the current study.
Population characteristics	In the TCGA cohort, the age of patients ranged from 10 to 89, with a median age of 59. Similarly, in the CPTAC cohort, the age of patients ranged from 24 to 80, with a median age of 59. In the spatial transcriptomics cohort, the age ranged from 34 to 81, with a median age of 63.
Recruitment	The current study does not involve the recruitment of any new human research subjects.
Ethics oversight	No ethical approval was required for the data included in the current study.

Note that full information on the approval of the study protocol must also be provided in the manuscript.

Field-specific reporting

Please select the one below that is the best fit for your research. If you are not sure, read the appropriate sections before making your selection.

☒ Life sciences ☐ Behavioural & social sciences ☐ Ecological, evolutionary & environmental sciences

For a reference copy of the document with all sections, see nature.com/documents/nr-reporting-summary-flat.pdf

Life sciences study design

All studies must disclose on these points even when the disclosure is negative.

Sample size	The sample size (listed in Supplementary Table 1) is determined based on data availability, taking into consideration the data exclusion criteria described below.
Data exclusions	As described in the "Results" section, since the absolute size for the resected tumor region varied across patients, the downstream analysis of tissue compositions may lead bias to tumors with large resections. To overcome this potential sampling bias, we implemented two strategies. First, we ranked the tumors in each cohort based on their number of patches (indicating tissue size) and removed the bottom 5% tumors with the smallest number of patches. Second, we included gender, age, IDH status, and tissue size as covariates in our Cox regression analysis. Following this rigorous filtering strategy, we obtained a final set of 693 slides (n = 312 patients) in the TCGA cohort and 227 slides (n = 98 patients) in the CPTAC cohort.
Replication	In the current study, we aimed to ensure the reproducibility of our findings by testing them across several independent patient cohorts. Significant associations discovered in one patient cohort were validated in one or more additional patient cohorts. To assess the performance of our models for transcriptional subtype classifications, we performed internal cross-validation using data from the spatial transcriptomics cohort, and further tested the model in the IvyGap and TCGA cohorts. In addition, to determine whether the significant associations between spatial cellular organization and patient prognosis discovered in the TCGA cohort (n = 312 patients) can be reproduced, we validated them in the CPTAC cohort (n = 98 patients).

Randomization	This is an observational study where randomization is not applicable.
Blinding	Blinding is not relevant to this study since it is an observational study that relies on automated computational methods for generating results, eliminating the need for subjective evaluation or any human derived labels.

Reporting for specific materials, systems and methods

We require information from authors about some types of materials, experimental systems and methods used in many studies. Here, indicate whether each material, system or method listed is relevant to your study. If you are not sure if a list item applies to your research, read the appropriate section before selecting a response.

Materials & experimental systems

n/a	Involved in the study
☒	☐ Antibodies
☒	☐ Eukaryotic cell lines
☒	☐ Palaeontology and archaeology
☒	☐ Animals and other organisms
☒	☐ Clinical data
☒	☐ Dual use research of concern

Methods

n/a	Involved in the study
☒	☐ ChIP-seq
☒	☐ Flow cytometry
☒	☐ MRI-based neuroimaging
