# Supplementary material for: Spatial cellular architecture predicts prognosis in glioblastoma
Source: Nat Commun. 2023 Jul 11;14:4122. doi: 10.1038/s41467-023-39933-0 (PMC10336135; doi:10.1038/s41467-023-39933-0)
Supplement: Supplementary file 2 — Peer Review File [file 41467_2023_39933_MOESM2_ESM.pdf]

nature portfolio

Peer Review File

Spatial Cellular Architecture Predicts Prognosis in Glioblastoma

Open Access This file is licensed under a Creative Commons Attribution 4.0 International License, which permits use, sharing, adaptation, distribution and reproduction in any medium or format, as long as you give appropriate credit to the original author(s) and the source, provide a link to the Creative Commons license, and indicate if changes were made. In the cases where the authors are anonymous, such as is the case for the reports of anonymous peer reviewers, author attribution should be to 'Anonymous Referee' followed by a clear attribution to the source work. The images or other third party material in this file are included in the article's Creative Commons license, unless indicated otherwise in a credit line to the material. If material is not included in the article's Creative Commons license and your intended use is not permitted by statutory regulation or exceeds the permitted use, you will need to obtain permission directly from the copyright holder. To view a copy of this license, visit http://creativecommons.org/licenses/by/4.0/.

Reviewers' Comments:

Reviewer #1:
Remarks to the Author:
The manuscript Spatial Cellular Architecture Predicts Prognosis in Glioblastoma by Zheng et al. combines spatial transcriptomic data with imaging features on H&E sections. This type of work provides an very interesting and innovative approach and provides new insights into the use of H&E sections for pathology.

The manuscript directly dives into the annotation of histological features to the five transcriptional 'neighborhoods' defined previously by the Heiland group. While this analysis forms the backbone of the manuscript, I missed an analysis of the histological features by themselves. How much variability of the features is present within and between samples and how many imaging feature clusters are present? How much variability is seen between HE stainings? Is there any overlap of these feature clusters with the expression-based clusters.

The authors make use of glioblastomas, but no molecular definition is provided both in the training set (I believe that the training data contains IDH-mutated tumors) and in the test data (TCGA data likely contains (prognostic) entities that will no longer be diagnosed as glioblastoma). These samples should be removed from the analysis as the model is only as good as the input provided.

Data from the TCGA and CPTAC was used to explore correlation with survival. While this is interesting, the clinical annotation of TCGA is a but limited and therefore should be treated with more caution. Analysis of the degree of centrality suffers the same limitation in survival data.

Discussion is mainly a recap on the obtained results. It does not contain a discussion on study limitations, or comparative efforts such as the use of artificial intelligence for tumor diagnostics using H&E sections.

Normal tissue is recognized by the absence of copy number aberrations. However, there will be differences in tumor purity between spots and it is unclear how tumor purity affects classification. Line 321 states that "Genes related to low aggressiveness were primarily associated with neuronal development". This could be down to just a lower tumor purity.

Other points
1. The spatial scRNA data with images is important data to this dataset. Accession number to this data is not presented if I am correct - only a reference to [16]
2. Spots from the spatial transcriptome platform are more than once described as cells and interpretation seems as if they are single cells.
3. scRNA-seq and spatial transcriptomics (and H&E stainings) may be sensitive to batch effects.
4. The multivariate analysis used for table 1 only includes the five cell-type states. This should include other prognostic factors such as patient age and tumour size. Even in case the states are no longer significant, the findings of this manuscript are still important.
5. The number of slides in the training data is relatively low. It would help to see if there is other data that can be used.
6. Discussion line: "It has the potential to improve our understanding of how the spatial cellular organization contributes to disease susceptibility and progression." How can it contribute to susceptibility?

Reviewer #2:
Remarks to the Author:

This study produces two incredibly compelling results with innovative deep-learning methodology. While spatial transcriptomics and single-cell RNA sequencing has offered a new paradigm for data-driven inference in cancer, these authors demonstrate that - significantly - the same type of insights can be drawn from using far more abundanant/accessible histology data.

This work is innovative not only because it will provide an immediate alternate tool for data-driven prediction in the patient setting but because it suggests that there is significant predictive power to be found in image data that can be strongly correlated with more expensive data gathering approaches and that has not been fully mined.

The work is technically sound and exciting and I imagine will be of great interest both to those in cancer/GBM and to folks like me - who are interested in methods.

Minor Text:
- Missing periods after the equations in lines 598 and 604. The typesetting would be more readable if donein Latex or with larger exterior parentheses.
- Missing period after equation in line 635.
- Missing period after equation in line 663.

Reviewer #3:
Remarks to the Author:
Zheng et al. developed a deep learning model to predict spatially resolved transcriptional programs from histology images and an independent model to predict prognosis based on histology. By integrating these two models, they discovered survival-associated regional gene expression programs. Overall, the study is interesting and the models are potentially useful.

The main issue, however, is that the authors seem confused about “spots”, “cells”, and “transcriptional states/programs” throughout the manuscript. Their transcriptome training dataset from Ref. 16 is based on the 10X Visium platform, which lacks the single-cell resolution. Consequently, each spot may contain mixed cell types. Thus, the recurrent transcriptional programs they identify should not be attributed to a single cell type. When they describe their results and prepare figures, in most cases they shouldn’t use “cells”, but should have used “spots” or “transcriptional states/programs” instead.

Additional comments:
1. Figure 1 is a re-analysis of a published spatial transcriptomic dataset (Ref. 16). When they performed cNMF, it is unclear how they filtered the malignant spots and whether the spatially weighted meta-module correlation was performed (the correlation needs to be corrected for spatial dependencies). Did they just follow Ref. 16, or use a different threshold to retain more spots with lower malignant cell contents? While they also identified five modules similar to Ref. 16, they named them differently. What’s the rationale for this? And what is the relationship between their modules and Ref. 16? Overall, the main text and methods lack critical details and comparison with Ref. 16.
2. The training dataset is based on IDHwt GBM, while the testing samples may include IDHmut GBM. Do the models perform differently on these two GBM types? It is also an important factor to consider/stratify when performing survival-related analysis.
3. The authors need to be careful about their interpretation of the “hybrid state”. The hybrid state could result from a mixture of cells from different states, instead of single cells exhibiting hybrid states. Along this line, it is inappropriate to say “some tumors comprise only one or two malignant cell types”. In fact, individual spots in these tumors may contain more than two cell types. What they observed is that spots in these tumors are assigned to just one or two states.
4. Figure 2D: The in situ images for GFAP and Olig2 do not look very convincing.
5. Figure 2E, F: I’d like to see a side-by-side comparison between the transcriptional state composition

predicted by RNA-seq deconvolution and GBM-CNN for these two samples.
6. Tissue samples in Ref. 16 were freshly collected and snap-frozen, while TCGA-GBM and CPTAC used FFPE samples. The histology could be quite different between these samples due to different sample preparation procedures. How did the authors address/account for this issue in testing their machine-learning model?
7. Figure. 4F, the inter-tumor heterogeneity of gene expression seems too high to call a consistent pattern.
8. The online website is useful for the community, but I didn’t see any description or tutorial to guide new users. I tested it with a local TIFF file (H&E for a GBM sample used for 10x Visium), and the tool returned a warning message "Unsupported or missing image file error".

Reviewer #4:
Remarks to the Author:
In this paper, the authors developed a deep learning model using histology images to predict spatially resolved transcriptional programs. Applying the model to glioblastoma (GBM) spatial transcriptomics data identified association between patient prognosis and spatial cell type distributions in tumors. The authors also trained a second deep learning model to predict prognosis from histology images, which helped identify survival-associated regional gene expression programs. The trained models were further wrapped into GBM360, a software that allows users to characterize the spatial cellular architecture of new GBM cases. The addressed question is relevant and interesting to the bioinformatics community, and the text is easy to read. However, the reviewer has the following concerns:

1. The authors used two 10x Visium spatial transcriptomics datasets and categorized each spot into one of six cell types – normal, NPC-like, OPC-like, reactive astrocyte (RA), MES-hypoxia, MES-like). However, each spot in a 10x Visum sample can contain multiple cells (typically 1-10, sometimes up to 30). Therefore, the spots contain a mixture of different cell types. It is better to perform deconvolution to estimate the cell type fractions in each spot instead of annotating each spot as one single cell type.
2. It is interesting that the classification performance of GBM-CNN for all the classes are very high except for RA and MES-hypoxia (Fig 2B). It seems that it is harder to distinguish between these two cell types than other pairs. The authors should discuss why this is the case.
3. The process of validating GBM-CNN predictions using bulk RNA-seq deconvolution results is problematic (Fig 2G-L). In this study, deconvolution was performed using the spatial transcriptomics cohort as the reference (using the signature gene expression matrix), whereas the reference’s cell types were predicted using GBM-CNN. Therefore, data leakage may be present, and even in this case, the correlations are moderately low. It will be more meaningful to validate GBM-CNN predictions by finding an external annotated GBM reference, matching its cell type labels to the authors labels, performing deconvolution using the external reference, and calculating the correlations between the deconvoluted cell type fractions and GBM-CNN results.
4. It is unclear whether the p-values in Tables 1-3 were adjusted for multiple testing. If not, some of the p-values are only marginally significant and may become insignificant once corrected.
5. The authors should discuss why TCGA and CPTAC results (Tables 1-3) are sometimes quite different in terms of significance and overall direction.
6. The illustrations of degree centrality (DC) and cluster coefficient (CC) in Fig 3A are hard to understand. Having four demo networks for high/low DC/CC of a certain cell type will be more informative.
7. In lines 257-258, the authors stated that “detailed analysis of cellular interactions showed that higher frequencies of interactions between…”. However, the interaction strength between two cell types was defined as “the number of edges between two malignant cell types divided by the total number of edges between all malignant cell types” (lines 661-662), where edges refer to the neighborhood relations between patches. It is inaccurate to state this as cell-cell interaction analysis because frequently being in another cell’s neighborhood does not necessarily indicate interaction.

8. It is unclear whether the authors performed three different sets of Cox regression analyses (Tables 1, 2, 3) using different sets of variables. If so, the authors should fit one Cox model including all the studied variables (proportion, DC, CC) and see if the results are still significant. The authors may also study the interactions between these variables to gain more insight into the underlying mechanism.
9. In the second deep learning model (line 285), the authors used TCGA as the training data and CPTAC as the testing data. Based on the previous sections, the analysis results on TCGA and CPTAC data are often different. Therefore, this choice of training and testing data will probably result in a training-testing data mismatch. The authors need to justify their selection, or match the training/testing data sources.
10. When I tested GBM360 using the example slide, the cell type distribution figure failed to fully load.

Minor issues/comments:
1. The scatterplots, box plots, and some of the clip arts are of much lower resolution compared with other subfigures.
2. There are minor typos and grammatical errors in the text. For example, line 502 should be “over 35%” instead of “over than 35%”.

We appreciate the time and effort invested by the reviewers in evaluating our manuscript. We have thoroughly addressed their critiques and significantly improved the quality of our manuscript. First, we have expanded our training cohort by including additional spatial transcriptomics data. This strengthens the robustness and generalizability of our image models. Second, we have performed deconvolution analysis to estimate cell-type-specific compositions within the gene expression spots. This allows us to account for the mixture of cell types and provides a more accurate interpretation of transcriptional heterogeneity in glioblastoma. Third, we have addressed concerns regarding the discrepancies of the training and validation cohorts by including additional patient characteristics, such as IDH status, age and gender, as covariates into our survival analysis. Additionally, we have clarified our statistical analysis of subtype interactions and greatly improved the quality and clarity of figure presentations. Please review our detailed point-by-point response below.

Reviewer #1, expertise in GBM biology and prognosis (Remarks to the Author):

The manuscript Spatial Cellular Architecture Predicts Prognosis in Glioblastoma by Zheng et al. combines spatial transcriptomic data with imaging features on H&E sections. This type of work provides a very interesting and innovative approach and provides new insights into the use of H&E sections for pathology.

We appreciate this reviewer for the overall positive comments in the innovation of our work. Please see our point-to-point response below.

Major points:

1. The manuscript directly dives into the annotation of histological features to the five transcriptional 'neighborhoods' defined previously by the Heiland group. While this analysis forms the backbone of the manuscript, I missed an analysis of the histological features by themselves. How much variability of the features is present within and between samples and how many imaging feature clusters are present? How much variability is seen between HE staining? Is there any overlap of these feature clusters with the expression-based clusters.

   We appreciate the valuable suggestion from this reviewer regarding the analysis of histological features. In response to this comment, we extracted several features from H&E-stained images, including the (1) the color values from each image channel, (2) histogram features, and (3) texture features ("Methods: Extraction of histological features"). The histogram features quantified histogram counts of color channel values, while the texture features characterized the different combinations of distance and angle between pixels. The combined feature vector had a dimension of 105 × 1. To identify clusters based on the image features, we standardized each feature and performed dimensional reduction and UMAP visualization. We found that the extracted image features varied across samples (Supplementary Fig.3c), and there were no strong correlations between these feature clusters and the gene expression-based clusters (i.e., transcriptional subtypes). However, when we performed the same analysis on the feature vector (2048 × 1) extracted from the last layer of

the ResNet50 module of GBM-CNN (Fig.2a), we observed a strong correlation between the resulting clusters and the transcriptional subtypes in the validation samples (Supplementary Fig.3d). These results indicated that GBM-CNN learned latent representations of the transcriptional subtypes beyond raw histological features.

2. The authors make use of glioblastomas, but no molecular definition is provided both in the training set (I believe that the training data contains IDH-mutated tumors) and in the test data (TCGA data likely contains (prognostic) entities that will no longer be diagnosed as glioblastoma). These samples should be removed from the analysis as the model is only as good as the input provided.

   We appreciate the reviewer for providing this comment. Both the training set (spatial transcriptomics) and the test set (TCGA) contained both IDH-wild type and IDH-mutated tumors. In the training set, approximately 82% (18/22) of the patients were IDH wild type and four patients were IDH mutant. In the test set, 92% (289/312) of samples were IDH wildtype, and the remaining 8% were IDH mutant. To address this reviewer's concern, we added a comprehensive summary table (Supplementary Table 1) that presents the molecular characteristics of the cohorts, including the distribution of IDH status. Furthermore, in our analysis of patient prognosis, we have included IDH status as a covariate in the survival analysis ("Methods": Survival analysis). By incorporating IDH status as a covariate, we aim to control for the potential confounding effects of IDH mutations on patient outcomes.

3. Data from the TCGA and CPTAC was used to explore correlation with survival. While this is interesting, the clinical annotation of TCGA is a but limited and therefore should be treated with more caution. Analysis of the degree of centrality suffers the same limitation in survival data.

   We agree with this reviewer that the clinical annotations for the TCGA and CPTAC cohorts are limited, distributed across multiple data platforms, and should be treated with cautions. To address this concern, we took great care in curating the clinical data from reliable and well-established sources. For the TCGA cohort, we utilized a standardized and high-quality clinical dataset from a comprehensive study that integrated patient endpoints for 33 different cancer types (*Liu et al. Cell, 2018*). Similarly, for the CPTAC cohort, we utilized the original clinical annotations from the corresponding study of this cohort (*Wang et al. Cancer Cell, 2021*). In the revised manuscript, we have included these references in the "Methods" section (*Survival analysis*). This increased transparency and allowed readers to access additional details about the clinical datasets utilized in our analysis.

4. Discussion is mainly a recap on the obtained results. It does not contain a discussion on study limitations, or comparative efforts such as the use of artificial intelligence for tumor diagnostics using H&E sections.

We appreciate this reviewer's comments. In the revised manuscript, we have included additional discussion of the limitations (Discussion: lines 454-467) and the relevant results from comparable studies (Discussion: lines 388-405).

5. Normal tissue is recognized by the absence of copy number aberrations. However, there will be differences in tumor purity between spots and it is unclear how tumor purity affects classification. Line 321 states that "Genes related to low aggressiveness were primarily associated with neuronal development". This could be down to just a lower tumor purity.

   We agree with this reviewer that the tumor purity may vary across different spots. To address this concern, we first implemented a computational method to infer tumor-cell content (i.e., tumor purity) at each spot based on the analysis of copy number alterations (CNAs) ("Methods: CNA inference and prediction of tumor cell content"). To infer CNAs, we used a separate cohort of normal brain tissues (n = 6 tissues from n = 3 patients) as a reference. Since tumor samples demonstrated broad CNAs across different chromosomes (Fig.1a and Supplementary Fig.1b), we first selected a signature CNA event that was shared across all spots within each tumor. The tumor cell content was then estimated based on the signature CNA event. For a given spot i, the tumor cell content C was defined as $C_i = [A_{CNVi} - 1]/[\max(A_{CNV}) - 1]$ if the signature was chromosomal gain and $C_i = [1 - A_{CNVi}]/[1 - \min(A_{CNV})]$ if the signature was chromosomal loss. At least three tumor signatures were used in each tumor, and the average results were obtained to ensure robust and unbiased estimation. Spots with $C > 0.2$ were considered malignant spots and retained for subsequent analysis.

   We next compared the tumor-cell content of malignant spots across different transcriptional subtypes. We did observe a slightly lower tumor cell content in spots classified as NPC-like and OPC-like compared to spots classified as MES-like or MES-hypoxia. However, the difference in average tumor cell content among these subtypes was less than 5% (data not shown). To further investigate this observation, we selected the top 70% of spots with the highest tumor cell content for each transcriptional subtype and compared them with each other. Our results showed there was no statistical significance among these selected spots. These findings indicated that a proportion of spots classified as NPClike or OPClike were located at the infiltrating border between the tumor and peripheral normal tissues, which agrees with the results from a recently published study (Venkataramani et al., *Cell*, 2022).

Other points:

6. The spatial scRNA data with images is important data to this dataset. Accession number to this data is not presented if I am correct - only a reference to [16].

   We appreciate the reviewer's suggestion. In the revised manuscript, we have expanded the spatial transcriptomics dataset beyond the previous mentioned reference (ref 16). To improve transparency, we have included Accession URLs for all spatial transcriptomics and single-cell

RNA-seq datasets used in this study as Supplementary Table 1. The table is referenced in the first paragraph of the "Results" section.

7. Spots from the spatial transcriptome platform are more than once described as cells and interpretation seems as if they are single cells.

   We appreciate the valuable comment from this reviewer. We agree that there are distinctions between "spots" versus "cells", since each spot contains multiple cells. To address this concern, we have taken two complimentary approaches. First, we have ensured that the correct terminology was used when referring to "spots". The identity of spots was now described as "transcriptional subtype" instead of "cells". Second, we have incorporated deconvolution analysis to estimate the cell-type composition within each spot. To accomplish this, we used data from single-cell RNA-seq as references, and we aligned the cell types identified from single-cell RNA-seq to the spatial transcriptomics data (Fig.1g and "Methods: Align single-cell RNA-seq data to spatial transcriptomics"). Our results indicated that, in most spots, the dominate tumor cell type accounted for over 70% of all tumor cells. Notably, approximately 30% of the spots consisted exclusively of one tumor cell type (Fig.1h). These data demonstrated that the tumor cell composition was relatively homogenous within each individual spot. Please see our response to Reviewer #4 for more details.

8. scRNA-seq and spatial transcriptomics (and H&E stainings) may be sensitive to batch effects.

   We agree with this reviewer that scRNA-seq, spatial transcriptomics and H&E stainings are sensitivity to batch effects. To address this concern, we have included additional technical procedures into our data preprocessing to take account of the batch effect ("Methods"). For both spatial transcriptomics data and scRNA-seq data, we performed normalization and variance stabilization using regularized negative binomial regression (Hafemeister et al., 2019). We regressed out percentages of mitochondria-expressed genes per spot/cell and effects from cell cycles ("Methods": Preprocessing of the spatial transcriptomics data). These procedures allowed us to remove the influence of technical variances from downstream analyses while preserving biological heterogeneity.

   To correct the batch effect of H&E staining colors across samples, we performed stain normalization using StainTools (https://github.com/Peter554/StainTools) ("Methods: Image processing and data augmentation"). For this purpose, we randomly selected 20 histology images from the TCGA-GBM cohort as references, and we normalized the stain colors of each image of the spatial transcriptomics to match those of each reference. The average image features derived from all references were used for further analysis.

9. The multivariate analysis used for table 1 only includes the five cell-type states. This should include other prognostic factors such as patient age and tumour size. Even in case the states are no longer significant, the findings of this manuscript are still important.

Following the suggestions made from this reviewer, we have included gender, age, IDH status, and tumor size as covariates in our Cox regression analysis ("Methods": Survival analysis). We have updated the results presented in Figure 3, Tables 1 and 2 to reflect the changes.

10. The number of slides in the training data is relatively low. It would help to see if there is other data that can be used.

    To address this comment, we have expanded the spatial transcriptomics dataset by including 6 additional GBM samples from a recent study (Ren et al. Nat Commun, 2023). Additionally, we included 1 sample from the 10X Genomics dataset. Detailed information about these datasets, including Accession URLs, can be found in Supplementary Table 1.

11. Discussion line: "It has the potential to improve our understanding of how the spatial cellular organization contributes to disease susceptibility and progression." How can it contribute to susceptibility?

    We appreciate the reviewer for capturing this point. Since the current study focused on investigating the spatial cellular organization in tumor tissues, "progression" is a more relevant term than "susceptibility." We have removed the word "susceptibility" in our revised manuscript.

Reviewer #2, expertise in ST, computational methods and deep learning (Remarks to the Author):

This study produces two incredibly compelling results with innovative deep-learning methodology. While spatial transcriptomics and single-cell RNA sequencing has offered a new paradigm for data-driven inference in cancer, these authors demonstrate that - significantly - the same type of insights can be drawn from using far more abundant/accessible histology data.

This work is innovative not only because it will provide an immediate alternate tool for data-driven prediction in the patient setting but because it suggests that there is significant predictive power to be found in image data that can be strongly correlated with more expensive data gathering approaches and that has not been fully mined.

The work is technically sound and exciting, and I imagine will be of great interest both to those in cancer/GBM and to folks like me - who are interested in methods.

We appreciate this reviewer for the overall positive comments in the innovation, significance and the technical aspects of our work. Please see our point-to-point response below.

Minor Text:

1. Missing periods after the equations in lines 598 and 604. The typesetting would be more readable if done in Latex or with larger exterior parentheses.

Thanks for the careful reading of our manuscript. We have added periods after these two equations.

2. Missing period after equation in line 635.

Thanks! We have added a period after this equation.

3. Missing period after equation in line 663.

Thanks! We have added a period after this equation.

Reviewer #3, expertise in ST and GBM (Remarks to the Author):

Zheng et al. developed a deep learning model to predict spatially resolved transcriptional programs from histology images and an independent model to predict prognosis based on histology. By integrating these two models, they discovered survival-associated regional gene expression programs. Overall, the study is interesting, and the models are potentially useful.

We appreciate this reviewer for the overall positive comments in the innovation and significance of our work. Please see our point-to-point response below.

1. The main issue, however, is that the authors seem confused about “spots”, “cells”, and “transcriptional states/programs” throughout the manuscript. Their transcriptome training dataset from Ref. 16 is based on the 10X Visium platform, which lacks the single-cell resolution. Consequently, each spot may contain mixed cell types. Thus, the recurrent transcriptional programs they identify should not be attributed to a single cell type. When they describe their results and prepare figures, in most cases they shouldn’t use “cells”, but should have used “spots” or “transcriptional states/programs” instead.

   We appreciate the valuable comment from this reviewer. We agree that the 10X Visium platform lacks single-cell resolution, and each spot contains multiple cells. To address this concern, we have taken two complimentary approaches. First, we have ensured that the correct terminology was used when referring to “spots”. We now refer to spots as "transcriptional subtypes" to accurately represent the aggregated nature of the data. In addition, we have incorporated deconvolution analysis to estimate cell-type composition within each spot. To accomplish this, we used data from single-cell RNA-seq as references, and we aligned the cell types identified from single-cell RNA-seq to the spatial transcriptomics data (Fig.1g and “Methods: Align single-cell RNA-seq data to spatial transcriptomics”).

The results from our deconvolution analysis indicated that each spot was predominately occupied by one tumor cell type. In most spots, the dominate tumor cell type accounted for over 70% of all tumor cells (Fig.1h). Notably, approximately 30% of the spots consisted exclusively of one tumor cell type (Fig.1h). However, we also observed that tumor cells were often mixed with immune cells, such as T cells and macrophages, within a spot (Figs. 1i-j). Taking into consideration the presence of mixed cell populations, we have updated our image model based on the results from the deconvolution analysis (Fig.2). The refined model aimed at predicting the dominant tumor cell type at each spot/patch while treating immune cells as independent labels. As a result, we have updated our analysis for prognosis using the new model, as presented in Fig.3 and Tables 1 and 2.

Additional comments:

2. Figure 1 is a re-analysis of a published spatial transcriptomic dataset (Ref. 16). Overall, the main text and methods lack critical details and comparison with Ref. 16.

    (1) When they performed cNMF, it is unclear how they filtered the malignant spots and whether the spatially weighted meta-module correlation was performed (the correlation needs to be corrected for spatial dependencies). Did they just follow Ref. 16, or use a different threshold to retain more spots with lower malignant cell contents?

We appreciate this valuable comment from the reviewer regarding our methods for identifications of malignant spots. To address this concern, we implemented a computational method to infer tumor purity at each spot based on the analysis of copy number alterations (CNAs) ("Methods: CNA inference and prediction of tumor cell content"). To infer CNAs, we used a separate cohort of normal brain tissues (n = 6 tissues from n = 3 patients) as a reference. Since tumor samples demonstrated broad CNAs across different chromosomes, we first selected a signature CNA event that was shared across all spots within each tumor. The tumor cell content was then estimated based on the signature CNA event. For a given spot i, the tumor cell content C was defined as $C_i = [A_{CNVi} - 1]/[\max(A_{CNV}) - 1]$ if the signature was chromosomal gain and $C_i = [1 - A_{CNVi}]/[1 - \min(A_{CNV})]$ if the signature was chromosomal loss. At least three tumor signatures were used in each tumor, and the average results were obtained to ensure robust and unbiased estimation. Spots with $C > 0.2$ were considered malignant spots and retained for subsequent analysis.

We did not perform spatially weighted correlations between meta-gene modules. In the study from Ravi et al. (Ref 16.), the spatially weighted correlation analysis was employed to reduce the number of meta-gene modules and for detecting recurrent modules across the patients. However, our approach differs from their approach in that we used cNMF (consensus non-negative matrix factorization) to identify the recurrent gene modules. Although the cNMF process did not involve spatially weighted correlations, we observed that the cNMF modules were already spatially coordinated (Fig.1f). Furthermore, we demonstrated that these cNMF modules showed spatial correlation with published modules derived from the study by Ravi et al. (Ref 16.) and single-cell RNA-seq studies (see our response to the comment below).

(2) While they also identified five modules similar to Ref. 16, they named them differently. What's the rationale for this? And what is the relationship between their modules and Ref. 16?

There were two main reasons why we named the modules differently from those in Ref. 16 (Ravi et al.). First, in the revised manuscript, we expanded the spatial transcriptomics dataset by including additional samples from new studies (Supplementary Table 1). Second, we used cNMF to identify the meta-gene modules, which differs from the method used in Ref. 16 ("Methods": Consensus non-negative matrix factorization (cNMF)).

To assess how the cNMF modules were related to published gene expression modules, we added spatially weighted correlation analysis (Figs.1c-d). We first compared each cNMF module to the modules defined in a spatial transcriptomics study by Ravi et al. (ref 16). As expected, we observed strong correlations between the nmf.NPC and nmf.OPC modules with the Regional.NPC and Regional.OPC modules, respectively (Fig.1c, $P < 0.001$). The nmf.RA correlated with both the Radial.Glia (P < 0.001) and Reactive.Immune (P < 0.01) modules. This overlap was expected due to the close relationship discovered between the Radial.Glia and Reactive.Immune modules in the original study. The nmf.MES-like and nmf.MES-hypoxia modules were significantly correlated with the Reactive.Immune and Reactive.Hypoxia module, respectively (Fig.1C).

To provide additional validation of our modules, we further compared them to those defined from a single-cell RNA-seq study by Neftel et al. (ref. 5). The nmf.NPC, nmf.OPC, and nmf.RA modules were strongly correlated with the NPClike, OPClike and AClike modules, respectively (Fig.1d). Similarly, the nmf.MES-like and nmf.MES-hypoixa modules were strongly correlated with the MESlike1 and MESlike2 modules. Notably, the MESlike2 module identified by Neftel et al. was also enriched with hypoxia-response genes, demonstrating the strong relationship between the cNMF modules and those derived from single-cell RNA-seq.

(3) The training dataset is based on IDHwt GBM, while the testing samples may include IDHmut GBM. Do the models perform differently on these two GBM types? It is also an important factor to consider/stratify when performing survival-related analysis.

We appreciate the reviewer for providing this comment. Both the training set (spatial transcriptomics) and the test set (TCGA) contained a small portion of IDH-mutated tumors. In the training set, approximately 82% (18/22) of the patients were IDH wild type and four patients were IDH mutant. In the test set, 92% (289/312) of samples were IDH wildtype, and the remaining 8% were IDH mutant. To illustrate the distribution of IDH status in these cohorts, we have added a comprehensive summary table (Supplementary Table 1) that presents the molecular characteristics of the cohorts. As proposed by this reviewer, we have included IDH status as a covariate in the survival analysis ("Methods": Survival analysis). By incorporating IDH status as a covariate, we aim to control for the potential confounding effects of IDH

mutations on patient outcomes. Subsequently, we have updated the results presented Fig.3 and Tables 1 and 2.

(4) The authors need to be careful about their interpretation of the “hybrid state”. The hybrid state could result from a mixture of cells from different states, instead of single cells exhibiting hybrid states. Along this line, it is inappropriate to say “some tumors comprise only one or two malignant cell types”. In fact, individual spots in these tumors may contain more than two cell types. What they observed is that spots in these tumors are assigned to just one or two states.

We appreciate the valuable comment from this reviewer. Indeed, it is important to consider that a spot contains multiple cells, and it can be challenging to distinguish whether the observed transcriptomes represent a "hybrid state" or simply a mixture of cell types. To address this concern, we performed deconvolution analysis to estimate cell-type composition within each spot. We used data from single-cell RNA-seq as references, and we mapped the cell types identified from single-cell RNA-seq to the spatial transcriptomics data (Fig.1g and “Methods: Align single-cell RNA-seq data to spatial transcriptomics”). This allowed us to better capture the cell-type heterogeneity within the spots. Please see our response to Comment #1 from this reviewer.

We have taken the reviewer's suggestion and removed the figures that characterized the "hybrid state." Instead, we have incorporated updated results from the single-cell deconvolution analysis, which provide a better representation of the cell-type heterogeneity within the spots (Figs.1g-j).

(5) Figure 2D: The in situ images for GFAP and Olig2 do not look very convincing.

We appreciate the comment from this reviewer. The lower performance in our original study may result from a small training cohort in which some cell types were underrepresented. To address this, we have included additional spatial transcriptomics data from a recent study (Supplementary Table 1) to expand our training cohort. The training cohort now included 23 samples (n = 75,625 spots). This strengthens the robustness and generalizability of our image models. In addition, to better characterize the cellular compositions and improve the prediction performance, we have re-trained a new image model (Fig.2a) based on the results from the deconvolution analysis as mentioned in our response to Comment #1 from this reviewer.

We evaluated the performance of our improved image model on images from the IvyGap cohort. The updated results, as shown in Figs.2e-f, demonstrated a significant improvement in the correlation between the signals of gene signatures and the predicted transcriptional subtypes from our image model.

(6) Figure 2E, F: I'd like to see a side-by-side comparison between the transcriptional state composition predicted by RNA-seq deconvolution and GBM-CNN for these two samples.

In response to this comment, we added side-by-side comparisons between the transcriptional state compositions predicted by RNA-seq deconvolution and GBM-CNN for the two presented samples (Figs.2g-h). The results from these comparisons revealed a consistent pattern between the predictions obtained from the two independent modalities. Specifically, for the tumor presented in Fig.2g, the GBM-CNN model predicted a higher fraction of the AC-like subtype than the NPC-like and MES-hypoxia subtypes, which aligned with the predictions from RNA-seq deconvolution. Similarly, for the tumor in Fig.2h, the GBM-CNN model predicted a higher fraction of the NPC-like subtype compared to the AC-like or OPC-like subtypes, again consistent with the RNA-seq deconvolution results. These results further validated the performance of GBM-CNN in the external testing cohorts.

(7) Tissue samples in Ref. 16 were freshly collected and snap-frozen, while TCGA-GBM and CPTAC used FFPE samples. The histology could be quite different between these samples due to different sample preparation procedures. How did the authors address/account for this issue in testing their machine-learning model?

We appreciate the reviewer raising this concern regarding the potential differences in histology between freshly frozen samples used in Ref. 16 and the FFPE samples from TCGA and CPTAC cohorts. We implemented several processes to account for this issue during both the training and testing phases of our machine-learning model.

Firstly, we implemented color normalization during the training phase. Given the differences in H&E staining colors between the spatial cohort and the TCGA cohort, we performed stain normalization using StainTools (https://github.com/Peter554/StainTools) ("Methods: Image processing and data augmentation"). For this purpose, we randomly selected 20 histology images from the TCGA-GBM cohort as references, and we normalized the stain colors of each image from the spatial cohort to match those of each reference. The average image features derived from all references were used for further analysis.

Secondly, we incorporated several image augmentation strategies to account for the differences in technical variances during data collections ("Methods: Image processing and data augmentation"). This included random horizontal and vertical flipping in 50% of the time, as well as random adjustments of brightness (factor = 0.25), contrast (factor = 0.25), and saturation (factor = 0.25). The data augmentation procedures enhance the robustness and generalizability of our image models.

Finally, to test the performance of GBM-CNN in the TCGA cohort, we performed bulk RNA-seq deconvolution as an independent measurement of transcriptional state compositions, and we compared the results with the those predicted from histology images using GBM-CNN (Figs.2g-h-i and see our response to the last comment). The side-by-side comparisons revealed a consistent pattern between the predictions obtained from the two independent

modalities. These results validated the performance of GBM-CNN in predicting the transcriptional state composition in FFPE images.

(8) Figure. 4F, the inter-tumor heterogeneity of gene expression seems too high to call a consistent pattern.

We appreciate the reviewer for bringing this point into our attention. In our original study, we included all the malignant spots (n = 69, 647) into the heatmap, and we agree that there were variations for individual gene expression across different spots. It is importance to note that, despite the presence of variations, all the presented genes reached statistical significance when comparing the high-aggressive versus low-aggressive group (log2FC > 0.25 and adjusted *P* value < 0.01). In the revised manuscript, we only included the top 10,000 spots assigned with the highest and the lowest aggressive scores in our plot. This approach allowed us to focus on the most informative and representative spots for the analysis. Furthermore, we replaced the heatmap with a violin plot (Fig.4f) to provide a more comprehensive view of the distribution of gene expression between the high-aggressive and low-aggressive groups.

(9) The online website is useful for the community, but I didn't see any description or tutorial to guide new users. I tested it with a local TIFF file (H&E for a GBM sample used for 10x Visium), and the tool returned a warning message "Unsupported or missing image file error".

We appreciate this reviewer for spending time in testing our software. In response to these concerns, we have included additional descriptions of the GBM360 website and a tutorial for new users in our GitHub repository: https://github.com/gevaertlab/GBM360/blob/master/readme.md. To address the issue with the "Unsupported or missing image file" error, we have improved our software to extend support for both "**tif**" and "**tiff**" image formats. This adjustment ensures compatibility with images with different file extensions. We apologize for any inconvenience this may have caused and appreciate the reviewer for bringing it to our attention.

Reviewer #4, expertise in ST, computational methods and deep learning (Remarks to the Author):

In this paper, the authors developed a deep learning model using histology images to predict spatially resolved transcriptional programs. Applying the model to glioblastoma (GBM) spatial transcriptomics data identified association between patient prognosis and spatial cell type distributions in tumors. The authors also trained a second deep learning model to predict prognosis from histology images, which helped identify survival-associated regional gene expression programs. The trained models were further wrapped into GBM360, a software that allows users to characterize the spatial cellular architecture of new GBM cases. The addressed question is relevant and interesting to the bioinformatics community, and the text is easy to read. However, the reviewer has the following concerns:

We appreciate this reviewer for the detailed reading of our manuscript and the overall positive comments in the significance and text structure of our work. Please see our point-to-point response below.

1. The authors used two 10x Visium spatial transcriptomics datasets and categorized each spot into one of six cell types – normal, NPC-like, OPC-like, reactive astrocyte (RA), MES-hypoxia, MES-like). However, each spot in a 10x Visum sample can contain multiple cells (typically 1-10, sometimes up to 30). Therefore, the spots contain a mixture of different cell types. It is better to perform deconvolution to estimate the cell type fractions in each spot instead of annotating each spot as one single cell type.

   We agree with this reviewer that the 10X Visium platform lacks single-cell resolution, and each spot contains multiple cells. Following the suggestion from this reviewer, we have incorporated deconvolution analysis to estimate cell-type composition of each spot and updated our image model accordingly. To perform the deconvolution, we used data from single-cell RNA-seq as references and mapped the cell types identified from single-cell RNA-seq to the spatial transcriptomics data (Fig.1g and "Methods: Align single-cell RNA-seq data to spatial transcriptomics").

   The results from our deconvolution analysis indicated that each spot was predominately occupied by one tumor cell type. In most spots, the dominate tumor cell type accounted for over 70% of all tumor cells (Fig.1h). Notably, approximately 30% of the spots consisted exclusively of one tumor cell type with approximately 30% of the spots exclusively containing a single cell type (Fig.1h). However, we also observed that tumor cells were often mixed with other immune cells, such as T cells and macrophages, within a spot (Figs.1i-j). Taking into consideration the presence of mixed cell populations, we have updated our image model based on the results from the deconvolution analysis (Fig.2). The refined model aimed at predicting the dominant tumor cell type at each spot/patch while treating immune cells as independent labels. As a result, we have updated our analysis for prognosis using the new image model, as presented in Fig.3 and Tables 1 and 2.

2. It is interesting that the classification performance of GBM-CNN for all the classes are very high except for RA and MES-hypoxia (Fig 2B). It seems that it is harder to distinguish between these two cell types than other pairs. The authors should discuss why this is the case.

   The lower performance in distinguishing the RA versus MES-hypoxia state was possibly due to the similarity between these two states or the existence of mixed cell types in a single spot. To better characterize the cellular compositions and improve the prediction performance, we have re-trained a new image model based on the results from the deconvolution analysis as mentioned in our response to the previous comment. This new model achieved better predicting performance in distinguishing the AC-like and the MES-like states (Fig.2b). This improvement indicates that our revised model better captured the underlying transcriptional heterogeneity within GBM and provides more reliable predictions within the spatial context.

3. The process of validating GBM-CNN predictions using bulk RNA-seq deconvolution results is problematic (Fig 2G-L). In this study, deconvolution was performed using the spatial transcriptomics cohort as the reference (using the signature gene expression matrix), whereas the reference's cell types were predicted using GBM-CNN. Therefore, data leakage may be present, and even in this case, the correlations are moderately low. It will be more meaningful to validate GBM-CNN predictions by finding an external annotated GBM reference, matching its cell type labels to the authors labels, performing deconvolution using the external reference, and calculating the correlations between the deconvoluted cell type fractions and GBM-CNN results.

   We appreciate the reviewer's careful evaluation of our manuscript. While we acknowledge the reviewer's concern regarding using the bulk RNA-seq deconvolution results to validate GBM-CNN, we respectfully disagree with the notion of data leakage. It is important to clarify that bulk RNA-seq and histology images are two distinct and independent modalities. The predictions of transcriptional subtypes from GBM-CNN were based solely on histology images, while the deconvolution analysis was based on bulk RNA-seq. GBM-CNN has no knowledge about the bulk RNA-seq data while making the predictions. Likewise, the deconvolution analysis of bulk RNA-seq was conducted without incorporating any information from the histology images.

   As mentioned by this reviewer, we used the top-scoring transcriptomes of each transcriptional state from the spatial transcriptomics as references to construct the signature gene expression matrix. This was done to ensure comparability between the transcriptomic subtypes inferred from GBM-CNN and the results obtained from bulk RNA-seq deconvolution. By using the same signature gene matrix to define transcriptomic subtypes, we aimed to establish a meaningful and valid comparison between results from the two modalities. If we were to use an "external annotated GBM reference", where the transcriptional subtype annotations differ from our own, we would face a problem of comparing disparate cell-type classifications.

In our study, we have included the IvyGap cohort as an additional external validation cohort to the TCGA cohort. We observed that tumor cells assigned with a specific transcriptional subtype from GBM-CNN had high messenger RNA expression levels (i.e., ground truth) for the corresponding transcriptional-state signatures (Figs.2e-f).

Additionally, in the revised the manuscript, we used data from single-cell RNA-seq as a reference to define the transcriptional subtype of spatial transcriptomics and to deconvolute bulk RNA-seq data from TCGA (see our response to Comment #1 from this reviewer). This has prevented any information flow between the TCGA and the spatial cohort.

4. It is unclear whether the p-values in Tables 1-3 were adjusted for multiple testing. If not, some of the p-values are only marginally significant and may become insignificant once corrected.

   We appreciate the reviewer for bringing this point to our attention. We have clarified in the "Methods" section (*Survival analysis*) that the *P* values were adjusted for multiple testing using the Benjamini-Hochberg method.

5. The authors should discuss why TCGA and CPTAC results (Tables 1-3) are sometimes quite different in terms of significance and overall direction.

   We appreciate the comment from this reviewer. The observed differences between the two cohorts can be attributed to two major factors. Firstly, the number of patients from CPTAC (n = 98) was much smaller than the number in the TCGA cohort (n = 312). This difference in sample size can lead to variations in the statistical significance observed between the cohorts. Secondly, we observed the presence of technical variations in the colors of H&E staining between the cohorts, which can impact the analysis. To address this issue, we implemented a stain normalization step using the StainTools library (https://github.com/Peter554/StainTools) as described in the "Methods: Image processing and data augmentation" section of our manuscript. Specifically, we randomly selected 20 histology images from the TCGA-GBM cohort as references, and we normalized the stain colors of each image from the CPTAC cohort to match those of each reference. The average image features derived from all references were then used for subsequent analysis. Following the stain normalization process, we observed that the two cohorts demonstrated more consistent patterns in Tables 1-2. This normalization step helped to mitigate the technical variations in staining colors and enhanced the comparability of the results between the CPTAC and TCGA cohorts.

6. The illustrations of degree centrality (DC) and cluster coefficient (CC) in Fig 3A are hard to understand. Having four demo networks for high/low DC/CC of a certain cell type will be more informative.

   In response to this reviewer's comment, we have made significant improvement in the characterization and demonstration of cellular interactions as presented in Fig.3. We

recognized degree centrality (DC) and clustering coefficient (CC) are counter measurements, where a cell type with high DC typically has low CC in the same sample, and vice versa. To avoid redundancy and improve clarity, we decided to focus solely on the analysis of CC and removed the characterization of DC. In order to provide a clearer demonstration of the cellular organization, we included two representative slides with high CC (Figs.3d-e) and another two with low CC of the AC-like subtypes (Figs.3f-g) in the revised manuscript.

To enhance the visualization of spatial cellular interactions, we generated abstractive networks based on the predicted cellular maps (Figs.3d-g and "Methods"). These networks provide a high-level illustration of the cellular interactions within the spatial neighborhood of the corresponding samples. By juxtaposing these abstractive graphs alongside the histology images, we aimed to improve the visualization and understanding of the cellular organization.

7. In lines 257-258, the authors stated that "detailed analysis of cellular interactions showed that higher frequencies of interactions between…". However, the interaction strength between two cell types was defined as "the number of edges between two malignant cell types divided by the total number of edges between all malignant cell types" (lines 661-662), where edges refer to the neighborhood relations between patches. It is inaccurate to state this as cell-cell interaction analysis because frequently being in another cell's neighborhood does not necessarily indicate interaction.

   We appreciate the comment from this reviewer. By using "edges", we meant "direct interactions" between patches. We have added clarifications to this in both the "Results" and "Methods" sections (lines 293 and 709).

8. It is unclear whether the authors performed three different sets of Cox regression analyses (Tables 1, 2, 3) using different sets of variables. If so, the authors should fit one Cox model including all the studied variables (proportion, DC, CC) and see if the results are still significant. The authors may also study the interactions between these variables to gain more insight into the underlying mechanism.

   Yes, that is correct. We assessed the effect of transcriptional subtype proportions and clustering coefficient (CC) on prognosis independently. To address the concern from this reviewer, we have included subtype proportions as covariates in our analysis for CC, as described in our revised manuscript (Results: lines 295-297). By doing so, we aimed to account for any potential confounding effects of cell-type proportions on the association between CC and prognosis. The results of our analysis showed that higher CC of AC-like subtype was significantly associated with a worse prognosis, even after adjusting for the subtype proportions (Table 2). These results highlighted the value of spatial relationships rather than abundance alone in predicting prognosis. As mentioned in our response to Comment #6 from this reviewer, since degree centrality (DC) and clustering coefficient (CC) are counter measurements, we have removed the characterization of DC in the revised manuscript.

9. In the second deep learning model (line 285), the authors used TCGA as the training data and CPTAC as the testing data. Based on the previous sections, the analysis results on TCGA and CPTAC data are often different. Therefore, this choice of training and testing data will probably result in a training-testing data mismatch. The authors need to justify their selection, or match the training/testing data sources.

   We appreciate the reviewer's attention to the observed differences between the CPTAC and TCGA cohorts. As we described in our response to Comment #5 from this reviewer, one critical factor underlying the observed differences between the two cohorts was technical variation in the colors of H&E staining between them. To address this issue, we implemented a stain normalization step using the StainTools library (https://github.com/Peter554/StainTools) as described in the "Methods: Image processing and data augmentation" section of our manuscript. Specifically, we randomly selected 20 histology images from the TCGA cohort as references, and we normalized the stain colors of each image from the CPTAC cohort to match those of each reference. The average image features derived from all references were then used for subsequent analysis. Following the stain normalization process, we observed that the two cohorts demonstrated more consistent patterns as demonstrated in Tables 1-2. This normalization step helped to mitigate the technical variations in staining colors and enhanced the comparability of the results between the CPTAC and TCGA cohorts.

10. When I tested GBM360 using the example slide, the cell type distribution figure failed to fully load.

   We appreciate this reviewer for taking the time to test our software. We would like to clarify that by default, GBM360 operates in "test" mode, where only a subset of patches (n = 1,000) from the entire image are predicted and visualized. This limitation is in place to provide users with a quick preview of the results. However, users have the option to switch from the test mode to a "complete" mode using the drop-down list located above the submission button. We would like to acknowledge that the complete mode may currently experience longer processing times due to computational constraints. To address this, we are actively seeking GPU support to optimize the speed and ensure that the full image can be predicted in a timely manner.

Minor issues/comments:

11. The scatterplots, box plots, and some of the clip arts are of much lower resolution compared with other subfigures.

   We appreciate the reviewer for pointing out the discrepancy in image resolution between certain subfigures in our manuscript. In response to this feedback, we have taken steps to replace any low-resolution images with higher-resolution versions. In addition, we are pleased to work with the editors to ensure every figure meets the publication's quality standards.

12. There are minor typos and grammatical errors in the text. For example, line 502 should be “over 35%” instead of “over than 35%”.

    We appreciate the reviewer for the careful reading of our manuscript. We have thoroughly reviewed the manuscript and have made the necessary corrections to address the minor typos and grammatical errors.

Reviewers' Comments:

Reviewer #1:
Remarks to the Author:
The authors have addressed my comments adequetely and I have no further points. The manuscript is very interesting and the approach innovative.

Reviewer #3:
Remarks to the Author:
The authors have adequately addressed all my concerns, and I'd congratulate them on this interesting work.

Reviewer #4:
Remarks to the Author:
The authors have satisfactorily addressed the reviewer's comments.

REVIEWERS' COMMENTS

Reviewer #1 (Remarks to the Author):

The authors have addressed my comments adequately and I have no further points. The manuscript is very interesting and the approach innovative.

We appreciate the time and input from this reviewer. Thank you!

Reviewer #3 (Remarks to the Author):

The authors have adequately addressed all my concerns, and I'd congratulate them on this interesting work.

We appreciate the time and input from this reviewer. Thank you!

Reviewer #4 (Remarks to the Author):

The authors have satisfactorily addressed the reviewer's comments.

We appreciate the time and input from this reviewer. Thank you!
